# Pathway-dependent supramolecular polymerization by planarity breaking†

**DOI:** 10.1039/d4sc02499k

**Published:** 2024-06-11

**Authors:** Rasitha Manha Veedu, Zulema Fernández, Nils Bäumer, Antonia Albers, Gustavo Fernández

**Affiliations:** a Universität Münster, Organisch-Chemisches Institut Corrensstraße 36 Münster 48149 Germany fernandg@uni-muenster.de

## Abstract

In controlled supramolecular polymerization, planar π-conjugated scaffolds are commonly used to predictably regulate stacking interactions, with various assembly pathways arising from competing interactions involving side groups. However, the extent to which the nature of the chromophore itself (planar *vs.* non-planar) affects pathway complexity requires clarification. To address this question, we herein designed a new BOPHY dye 2, where two oppositely oriented BF_2_ groups induce a disruption of planarity, and compared its supramolecular polymerization in non-polar media with that of a previously reported planar BODIPY 1 bearing identical substituents. The slightly non-planar structure of the BOPHY dye 2, as evident in previously reported X-ray structures, together with the additional out-of-plane BF_2_ group, allow for more diverse stacking possibilities leading to two fiber-like assemblies (kinetic 2A and thermodynamic 2B), in contrast to the single assembly previously observed for BODIPY 1. The impact of the less rigid, preorganized BOPHY core compared to the planar BODIPY counterpart is also reflected in the stronger tendency of the former to form anisotropic assemblies as a result of more favorable hydrogen bonding arrays. The structural versatility of the BOPHY core ultimately enables two stable packing arrangements: a kinetically controlled antiparallel face-to-face stacking (2A), and a thermodynamically controlled parallel slipped packing (2B) stabilized by (BF_2_) F⋯H (*meso*) interactions. Our findings underscore the significance of planarity breaking and out-of-plane substituents on chromophores as design elements in controlled supramolecular polymerization.

## Abstract 2

We introduce planarity breaking as a new design strategy for controlled supramolecular polymerization.

## Graphical abstract

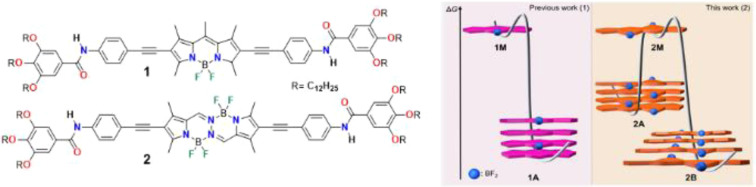

## Introduction

Self-assembled structures of π-conjugated chromophores have received considerable interest in recent decades due to their promising potential in various fields such as optoelectronics,^1,2^ sensing,^3,4^ bioimaging^5,6^ and light harvesting devices.^7^ Key properties of these supramolecular ensembles, such as charge transport^8^ or near infrared (NIR) emission,^9,10^ are coupled to the molecule's arrangement in the assembled state. Accordingly, gaining control over intermolecular association, packing and morphology is a crucial step towards optimizing functional properties.^8,11^ In this context, molecular design has become a powerful tool to tune (complex) energy landscapes in self-assembly, as evident by detailed analysis of a wide range of molecular building blocks.^12–38^ While various studies have revealed that the intermolecular interactions encoded in the monomer design largely govern the self-assembly outcome, predicting these interactions by molecular design is far from easy. For example, we recently found that the functionalization of dye molecules with bulky substituents may unexpectedly stabilize H-type face-to-face stacking interactions despite the significant steric hindrance, contrary to conventional expectations based on literature.^39^ Thus, there is still an urgent need for improved methods to predict structure–property relationships in self-assembly.

When considering the extensive literature on self-assembled π-conjugated systems, researchers often exploit the inherent planarity of π-conjugated scaffolds and dye molecules as a means to predictably control their stacking arrangements.^40,41^ Although much less studied, non-planar π-conjugated molecules also demonstrate extended self-assembly potential in solution, given appropriate functionalization.^42–44^ However, an unexplored aspect is whether the sole disruption of planarity in π-systems may originate multiple assembled states using the same building block. In this context, tetracoordinated organoboron dyes represent an ideal choice to investigate such effects, given that the typically used boron difluoride groups (BF_2_) are arranged out of plane and, thus, may induce molecular distortions. In addition to the well-known boron dipyrromethene (BODIPY) dyes,^45–48^ where the BF_2_ is linked to a dipyrromethene core, various analogous scaffolds, including azaBODIPYs,^12^ BODIHYs (hydrazones),^49,50^ boron-locked anilido pyridines,^51^ bis(borondifluoride)-8-imidazodipyrromethenes (BOIMPYs)^52^ and azaBOIMPYs,^53^ have been developed to leverage the photophysical properties and applications of this class of dyes. A particularly notable scaffold from the structural viewpoint is the BOPHY core (bis(difluoroboron)-1,2-bis((1*H*-pyrrol-2-yl)methylene)hydrazine), featuring two BF_2_ groups within a tetracyclic pyrrole-boron difluoride structure.^54^ Its structural resemblance to BODIPY, coupled with the non-planarity evident in its crystal structure,^54^ renders it an ideal candidate for exploring the impact of planarity disruption on complex supramolecular polymerization.

**Scheme 1:**
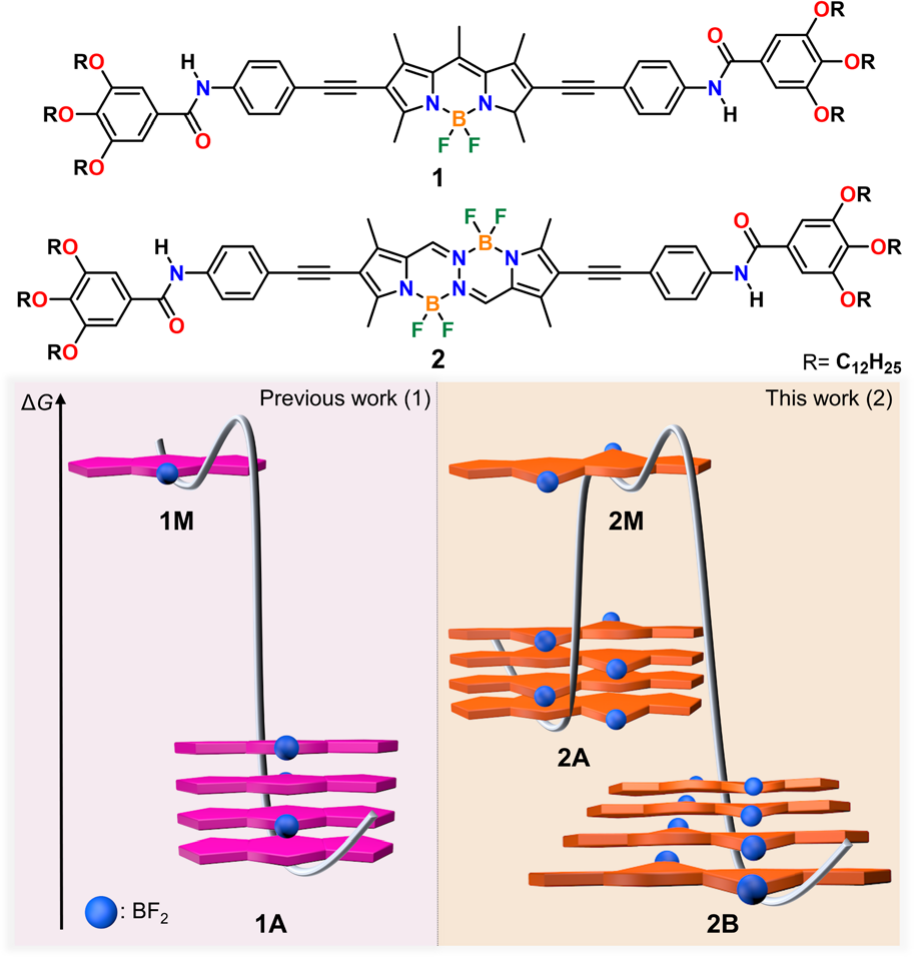
Molecular structures of model compound BODIPY (1) and BOPHY dye (2) and cartoon representation of their molecular packing modes along with the energy profiles associated with their self-assembly.

In this work, we demonstrate that planarity breaking enables different supramolecular polymerization pathways with distinct molecular arrangements. To this end, we designed a new π-extended BOPHY dye containing amide groups and solubilizing alkyl side chains (2) and compared its supramolecular polymerization in non-polar media with that of a previously reported BODIPY analogue (1) with identical substituents (Scheme 1). Detailed spectroscopic investigations in methylcyclohexane (MCH) revealed that the BOPHY derivative (2) exists as either of two assembled states (2A & 2B), unlike the BODIPY counterpart (1), which forms only one assembled state (1A).^39,55^ Interestingly, both 2A & 2B are described by a cooperative mechanism, which is reflected in the formation of elongated fibres for both assembled states, as imaged by atomic force microscopy (AFM) and scanning electron microscopy (SEM). In contrast, despite following the same aggregation mechanism, the corresponding BODIPY derivative (1) forms much shorter one-dimensional (1D) assemblies than the BOPHY compound (2). This may result from the higher rigidity of the planar BODIPY core compared to the BOPHY counterpart, which limits extended hydrogen bonding formation. Nuclear magnetic resonance (NMR) and Fourier-transform infrared (FTIR) spectroscopy along with theoretical calculations disclose amide–amide hydrogen bonds of similar strength for both 2A & 2B. However, the key difference is the packing mode: while H-type antiparallel face-to-face stacking interactions are observed for kinetic assembly 2A, the thermodynamic product 2B is stabilized by a parallel slipped packing and concomitant (BF_2_) F⋯H (*meso*) interactions. Therefore, the slightly non-planar structure of the BOPHY, together with the additional BF_2_ group, allow for more diverse stacking possibilities compared to the planar BODIPY dye. These findings underline the importance of planarity breaking and out-of-plane substituents on chromophores as design elements in controlled supramolecular polymerization.

## Results and discussion

### Synthesis and supramolecular polymerization

The target BOPHY derivative 2 was prepared following a synthetic procedure similar to that previously reported for the corresponding BODIPY derivative 1 (see ESI†).^55^ The major synthetic step includes the carbon–carbon cross coupling Sonogashira reaction between the diiodo BOPHY derivative (D, Fig. S1†) and the alkyne side fragment functionalized with dodecyloxy chains (E, Fig. S1†), affording 2 with 39% yield (Fig. S1†).

**Fig. 1:**
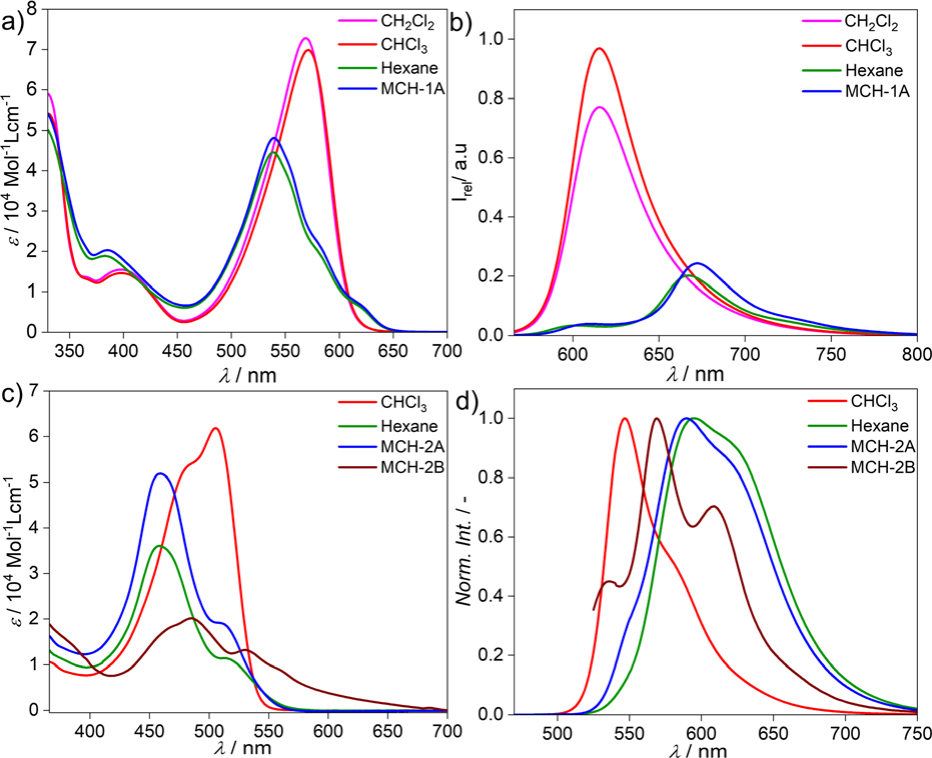
Solvent-dependent absorption studies of: (a) compound 1, (c) compound 2 and solvent-dependent fluorescence studies of: (b) compound 1, *λ*_exc_ = 510 nm and (d) compound 2, *λ*_exc_ = 470 nm at 298 K, *c* = 2 × 10^−5^ M.

The model BODIPY compound 1 has been thoroughly investigated in previous reports in terms of its supramolecular polymerization in non-polar solvents.^39,55^ This derivative was found to self-associate in MCH into 1D H-type stacks (1A) as single thermodynamic product. Irrespective of the experimental conditions (stirring, sonication, thermal or solvophobic quenching, cooling/heating and denaturation), 1 forms exclusively this self-assembled state 1A without traces of other kinetic products (Fig. 1a and b). Although 1A is formed *via* the cooperative mechanism, only short 1D assemblies are found in solution, which is attributed to increasing steric repulsion between the solubilizing chains hindering extended aggregate growth, as suggested by theoretical calculations.^56^ We argue that the modification of the BODIPY core may be an effective strategy to tune this behaviour towards more feasible elongated growth. To probe this hypothesis, the photophysical properties of the BOPHY derivative (2) were first investigated using solvent-dependent absorption and emission spectroscopy (Fig. 1c and d). At a concentration of 2 × 10^−5^ M, the absorption studies in moderately polar organic solvents like chloroform show spectral patterns that agree with a molecularly dissolved state (Fig. 1c), *i.e*. an absorption maximum at around 505 nm, corresponding to the S_0_ → S_1_ transition of the BOPHY chromophore,^55^ along with a shoulder at *λ*_max_ = 482 nm (Fig. 1c, see also Fig. S4a† for a comparison in multiple organic solvents). The corresponding photoluminescence studies of monomeric 2 in chloroform exhibit an emission maximum at ∼546 nm along with a red shifted shoulder at 586 nm (Fig. 1d & S4b†). This trend changes considerably if the system is investigated in non-polar solvents, such as MCH, hexane, heptane or dodecane. In these media, a hypsochromic shift of the absorption maximum to 458 nm is observed with respect to the molecularly dissolved state (Δ*λ* = 50 nm) (Fig. 1c & S4†). This self-assembled state will be termed from now on 2A. In emission studies, 2A is characterized by a sharp emission band at around 590 nm that is red-shifted compared to the molecularly dissolved state in chloroform (Δ*λ* = 44 nm). The observed spectral features of 2A bear close resemblance to those observed for 1 (1A), which are typical for a face-to-face (H-type) stacking of the BODIPY^55^ as well as the BOPHY dyes.^57^

Variable temperature UV-vis studies (VT UV-vis) at different concentrations and cooling rates were subsequently recorded to gain insights into the self-assembly mechanism of the BOPHY derivative 2 in MCH. Upon cooling a solution of 2 from 363 K to 263 K with a ramp rate of 1 K min^−1^, the absorption spectrum of the molecularly dissolved state (505 nm) shifts to lower wavelengths (458 nm), which can be attributed to the self-assembled species 2A (Fig. 3a & S5†). The formation of 2A was found to be independent of the cooling rate (Fig. S5†) and concentration (Fig. S6†), as also observed in VT emission studies (Fig. S7†), indicating that only one aggregate is obtained using thermal approaches. Plotting the degree of aggregation *α*_agg_*vs.* the temperature from the heating and cooling experiments under similar conditions does not reveal any thermal hysteresis (Fig. S8†). This indicates that no kinetically trapped products are formed during the thermally-induced monomer-to-aggregate transition. To confirm this further, we subjected the sample to more drastic changes in the experimental conditions. Both solvophobic quenching (rapid injection of monomeric solution of 2 into an excess of MCH) as well as thermal quenching (fast cooling of a hot monomeric solution of 2 in MCH to 273 K) resulted in identical spectral features (Fig. S9a and b†), which are characteristic for the formation of aggregate 2A. Hence, a single supramolecular species has been detected by thermal approaches, similar to what was found for the BODIPY counterpart 1.^55^

**Fig. 2:**
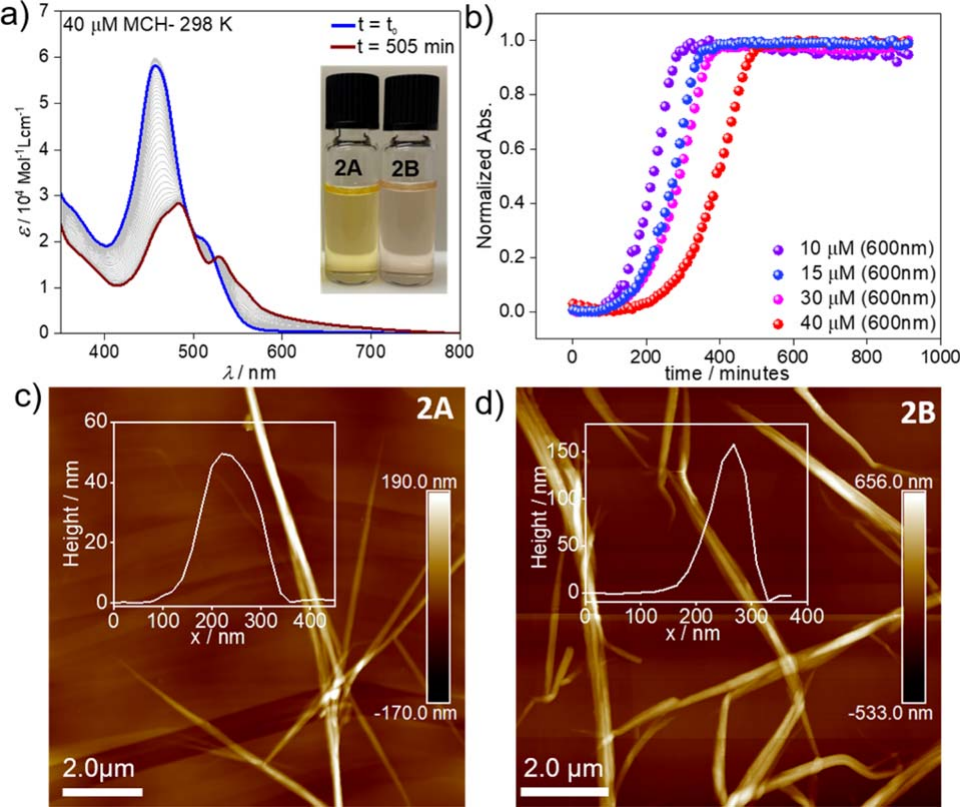
(a) Time-dependent evolution of assembly 2A into 2B in MCH (*c* = 4 × 10^−5^ M) at 298 K. (b) Plot of absorbance *vs.* time at *λ* = 600 nm using different concentrations (*c* = 10–40 μM) at 298 K. AFM images of 2A (c) and 2B (d) obtained upon drop-casting the corresponding solutions (*c* = 1 × 10^−5^ M) on HOPG.

However, dramatic differences in their time-dependent behaviour are witnessed for assemblies 1A and 2A. While 1A remains invariant over time due to its thermodynamic stability,^55^2A forms over the course of one day at room temperature an energetically more favourable species (2B) (Fig. 2a). This transformation can be further accelerated using mechanical agitation in the form of sonication (10–20 seconds). The new self-assembled state 2B is spectroscopically characterized by an absorption maximum centred at 484 nm (blue-shifted compared to the monomer) and a second, less intense red-shifted shoulder at 530 nm (Fig. 1c and 2a, brown spectrum). Spectral patterns with both H- and J-type characteristics, such as those of 2B, are in line with the formation of HJ aggregates, as proposed in the literature.^58,59^ The 2A → 2B transformation is accompanied by a colour change of the solution from yellow (2A) to light pink (2B) (inset of Fig. 2a). The new aggregated species (2B, *ϕ*_F_ = 12%) displays a lower photoluminescence quantum yield than 2A (*ϕ*_F_ = 87%), which can be rationalized in different ways. The fluorescence found in aggregate 2A might likely arise from the imperfect dye arrangement, possibly caused by a molecular scaffold that is not fully planar. Furthermore, we hypothesize that there could be a reduction in non-radiative decays due to the enhanced rigidity of the molecular chains in the π–π stacked aggregate.^60^ The lower quantum yield of 2B could be additionally explained by weakened excitonic coupling in the aggregated state compared to 2A.^61^

To elucidate the nature of the 2A → 2B transformation (competitive or consecutive pathways), kinetic experiments at multiple concentrations were performed in MCH (10–40 μM) (Fig. 2a and b). As depicted in Fig. 2a, the spectral characteristics of 2A are diminished over time as the spectral features of 2B become dominant. Monitoring this transformation over time discloses a decelerated 2A → 2B conversion upon increasing the concentration, which indicates that both assembled states 2A and 2B are formed directly from the monomer, *i.e*. they are competitive (see energy diagram in Scheme 1 and Fig. 2b & S10†).

**Fig. 3:**
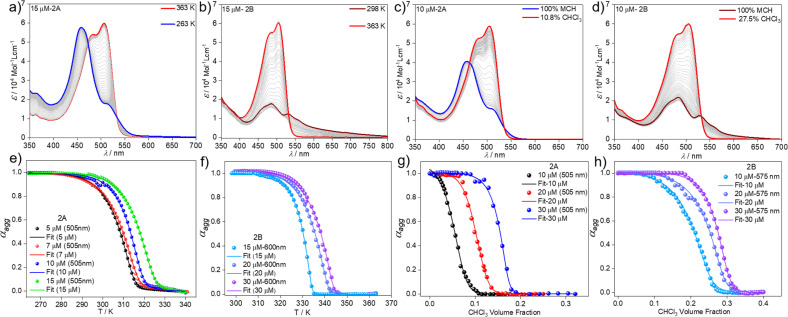
VT UV-vis studies in MCH (*c* = 1.5 × 10^−5^ M) with a cooling/heating rate of 1 K min^−1^ for 2A (a) and 2B (b). Plot of *α*_agg_*vs.* temperature monitored at *λ* = 505 nm for 2A (e) and at *λ* = 600 nm for 2B (f) and corresponding fits to the nucleation–elongation model. UV-vis studies at different MCH–CHCl_3_ ratios (*c* = 1 × 10^−5^ M) and 298 K for 2A (c) and 2B (d). Plot of *α*_agg_*vs.* CHCl_3_ volume fraction along with corresponding fits to the nucleation–elongation model for 2A (*λ* = 505 nm) (g) and 2B (*λ* = 575 nm) (h).

We next analysed the mechanism of formation of aggregates 2A and 2B by monitoring the absorption changes at a fixed wavelength against temperature during cooling experiments. In the case of aggregate 2A, thermodynamic analysis of the experimental data obtained at different concentrations revealed a cooperative supramolecular polymerization process (Fig. 3a and e). Fitting these cooling curves (1 K min^−1^) to the nucleation–elongation model^62^ gave an average Gibbs free energy of Δ*G* = −35.20 kJ mol^−1^ (Tables 1 and S1†). On the other hand, as the assembly 2B cannot be obtained by cooling experiments regardless of the cooling rate, we extracted the thermodynamic parameters from heating studies using a heating rate of 1 K min^−1^. These experiments revealed that the absorption pattern of the molecularly dissolved state gradually rises at the expense of the absorption features of 2B (Fig. 3b & S11†).

**Table 1:** Thermodynamic parameters obtained from VT UV-vis^62^ and denaturation experiments^63^ for aggregates 2A and 2B along with hydrogen bond distances calculated from theoretical calculations

Aggregate	Δ*G*_0_ (kJ mol^−1^) VT	Δ*G*_0_ (kJ mol^−1^) denaturation	C–H⋯F–B distances (Å)	N–H⋯O=C distances (Å)
2A	−35.20	−35.40	2.468, 3.595	2.105, 2.263
2B	−42.47	−40.68	1.999, 1.967	1.992, 2.074

The corresponding heating curves *vs.* temperature obtained at *λ* = 575 nm also exhibited a non-sigmoidal shape, suggesting a cooperative mechanism for aggregate 2B. Thermodynamic analysis of the plots at different concentrations (Fig. 3f) yielded a Δ*G* = −42.47 kJ mol^−1^ (Tables 1 and S2†). The lower Δ*G* value calculated for 2B compared to 2A demonstrates the superior stability of the former, which possibly arises from the more favourable chromophore packing arrangement. The high value of the nucleation penalty found for 2B compared to 2A suggests a higher cooperativity for the former (Tables S1 and S2†).^25^

Further information about the thermodynamic stability of the two assemblies was obtained by denaturation studies using CHCl_3_ as a denaturing solvent while monitored by UV-vis spectroscopy. Addition of aliquots of monomeric 2 in CHCl_3_ to the respective aggregate solutions of 2A (Fig. 3c & S12†) or 2B (Fig. 3d & S13†) at the same concentration leads to the disassembly of both aggregates directly to the monomeric species, further supporting the competitive nature of both pathways. Again, the cooperative model was employed to fit the denaturation measurements at different concentrations (Fig. 3g and h) for both aggregates.^63^ The thermodynamic parameters extracted from this experiment agree with the results obtained from the VT experiments and point to a higher stability of 2B (Δ*G* = −40.68 kJ mol^−1^) compared to aggregate 2A (Δ*G* = −35.40 kJ mol^−1^) (Table S3†) under the investigated conditions. The obtained energy difference between the two assembled states lies within the range of systems to be considered supramolecular polymorphs (approximately 10 kJ mol^−1^).^64–66,68^

Microscopy studies were performed to visualize the morphology of the assemblies after drop-casting their solutions onto highly oriented pyrolytic graphite (HOPG) or silicon wafer for AFM and SEM, respectively. Regardless of the employed substrate, the structures could be visualized as bundles of highly elongated fibre-like structures. AFM images of aggregate 2A showed the formation of needle-like morphologies with a height of *ca.* 50 nm and *ca.* 5–7 μm in length (Fig. 2c). In the case of 2B, elongated fibres can also be observed but with a height of approximately 150 nm and a length of *ca.* 3–5 μm (Fig. 2d). We infer from these results that both assemblies are significantly stabilized by hierarchical effects, leading to an efficient bundling of the structures.^67,69^ In the case of 2B, this behaviour is particularly pronounced (as evident from the increase in the height of the fibre bundles). We argue that this change in hierarchical interactions is driven by a decrease in the density of the solubilizing alkyl shell (*vide infra*), which allows the chains of neighbouring stacks to interdigitate more effectively in 2B. These observations are consistent with previous reports, which found an increased tendency to form hierarchical structures as a consequence of increased flexibility of solubilizing alkyl chains.^68^ In addition, polymerization processes driven by solvent–solute interactions have been demonstrated to follow a similar mechanism.^69,70^ The corresponding SEM studies (Fig. S18 & S19†) agree with the abovementioned results extracted from the AFM analysis, revealing less bundled fibres for 2A (Fig. S20†) and more clustered fibres for 2B (Fig. S21†) on the silicon wafer surface. TEM analysis (Fig. S22†) also reproduce the results from AFM and SEM.

**Fig. 4:**
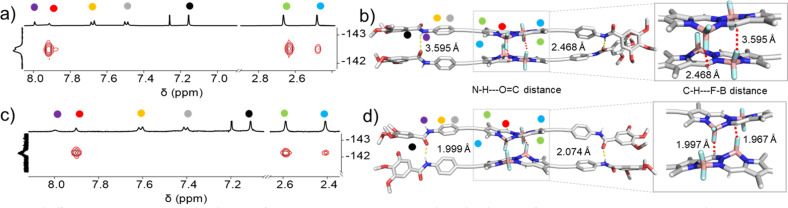
^1^H–^19^F HOESY 2D NMR studies of (a) 2A (*c* = 5 × 10^−3^ M, 70% MCH-d_14_ + 30% CDCl_3_ at 323 K) and (c) 2B (*c* = 5 × 10^−3^ M, 90% MCH-d_14_ + 10% CDCl_3_ at 323 K) illustrating the interactions of the *meso*-hydrogens of the BOPHY with the fluorine atoms. Corresponding molecular packing governed by amide–amide hydrogen bonding interactions as well as unconventional interactions between the *meso*-hydrogens and fluorines of the difluoride groups illustrated in (b) 2A and (d) 2B. The respective protons are also marked in the figure.

In order to rationalize the differences in molecular packing, FTIR measurements as well as ^1^H NMR studies were performed. For molecularly dissolved 2 in CHCl_3_ at 1 mM (Fig. S16†), we found N–H and C=O stretching bands at *ν*_N–H_ = 3432 cm^−1^ and *ν*_C=O_ = 1673 cm^−1^, respectively. The FTIR spectrum of aggregate 2A (1 mM in MCH, Fig. S17†) revealed the shifting of these bands to *ν*_N–H_ = 3278 cm^−1^ and *ν*_C=O_ = 1646 cm^−1^, indicating the existence of N–H⋯O=C hydrogen bonds. In the case of aggregate 2B (1 mM in MCH, Fig. S17†), a slightly lower value for the N–H stretching (*ν*_N–H_ = 3259 cm^−1^) was observed, while the C=O stretching band showed a similar frequency (*ν*_C=O_ = 1646 cm^−1^) when compared to 2A. The slightly lower value of the N–H stretching frequency band indicates the marginally stronger amide hydrogen bonding interactions for the thermodynamic product 2B,^71^ while the identical C=O frequencies might suggest the existence of defects in 2B. To further analyse the differences in packing of aggregates 2A and 2B, we performed 2D ^1^H–^19^F NMR spectroscopy (^1^H–^19^F HOESY NMR). We simultaneously monitored the 2D ^1^H–^19^F HOESY NMR spectra of 2A (70% MCH-d_14_ + 30% CDCl_3_) and 2B (90% MCH-d_14_ + 10% CDCl_3_) at 328 K (*c* = 5 mM) (Fig. 4a, c & S15†). Both aggregates revealed a strong correlation signal that corresponds to intermolecular interactions between the *meso*-hydrogens of the BOPHY core and the fluorine atoms connected to the boron. Additionally, both assemblies revealed a correlation between the methyl protons on the BOPHY core and the fluorine atoms, suggesting similar stabilizing interactions in both aggregates 2A and 2B, albeit with slightly different arrangements of the chromophores (Fig. 4a, c, S29 & S30†). Based on the similar 2D HOESY NMR experiment, we assume that both aggregates 2A and 2B may adopt a packing that maintains both types of hydrogen bonding interactions depending upon the feasible alignment of the BOPHY chromophore (parallel or antiparallel).

### Theoretical studies

**Fig. 5:**
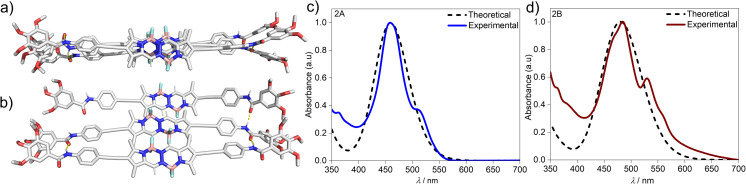
Geometry-optimized trimer structures of (a) aggregate 2A and (b) aggregate 2B obtained by DFT calculations (B3LYP/6-31(+)G(d,p)) and corresponding rCAM-B3LYP/6-31g(d,p) optimized absorption spectra of trimers of (c) 2A and (d) 2B.

To gain further insights on the packing modes of the aggregates, theoretical calculations—DFT, (B3LYP/6-31(+)G(d,p))^72,73^—were performed on monomers, dimers and trimers of 2A and 2B. In order to reduce the computational costs during the DFT calculations, we replaced the dodecyloxy side chains with methoxy groups. To corroborate the validity of our optimized structure, we calculated the absorption spectra for the monomers and trimers (rcam-B3LYP/6–31(+)G(d,p))^72,73^ and compared them with the experimental ones (Fig. 5c, d & S24†). The calculated absorption spectrum of a trimer of aggregate 2A shows a blue shift with respect to the monomeric species, which validates the proposed packing on the basis of the experimental data (Fig. 5a and c). The BOPHY chromophores within the trimer of 2A are arranged in a face-to-face antiparallel fashion, stabilized through N–H⋯OC hydrogen bonds as well as by weak interactions between the *meso*-hydrogens and the fluorines on the BOPHY core (Fig. 4b and 5a). Similarly, a trimer of 2B was optimized. The proposed packing displays the chromophores in a parallel arrangement, with the fluorine atoms of each BOPHY unit in the stack pointing in the same direction (Fig. 4d and 5b). Arranging the chromophores in a face-to-face fashion, as it was the case for the antiparallel packing of 2A, is not possible due to the steric hindrance of the out-of-plane fluorine atoms. As a result, the monomers are required to shift laterally along the short axis, retaining the amide N–H⋯OC hydrogen bonds as well the interactions between the *meso*-hydrogen of the core with the fluorines of the neighbouring molecule, as experimentally observed (Fig. 5b and d). The N–H⋯O=C amide hydrogen bond distances found for aggregate 2B (≈1.9–2.0 Å) are shorter than those of 2A (≈2.1–2.2 Å), suggestive of comparatively stronger hydrogen bonding interactions in the former aggregate (2B) (Table 1 & Fig. 4). These results agree with the FTIR measurements of both aggregates (Fig. S17†). Similarly, the C–H⋯F–B hydrogen bonding distance of aggregate 2A (≈2.4–3.5 Å) was also found to be greater than that of 2B (≈2.0 Å) (Table 1 & Fig. 4), again indicating comparatively stronger interactions within 2B. The predicted absorption spectra obtained for this trimer stack is in good agreement with the experimental trends (Fig. 5c and d). Thus, these studies suggest that the pathway complexity in the self-assembly of BOPHY derivative 2 arises from the different packing possibilities of the BOPHY chromophores due to the oppositely oriented BF_2_ groups, resulting in a loss of planarity.

## Conclusions

In summary, we have elucidated the supramolecular polymerization of a new class of chromophore (BOPHY), which is structurally analogous to the well-known BODIPY core, but it features an additional fused heterocycle and two oppositely oriented BF_2_ groups instead of one. These structural differences are responsible for the slight loss of planarity for the BOPHY core compared to the BODIPY counterpart, which greatly affects the overall supramolecular self-assembly. While the model BODIPY derivative 1 exists as only one type of supramolecular structure in non-polar media with face-to face (H-type) molecular packing, the new BOPHY derivative 2 forms two competitive supramolecular polymers. This can be explained by the additional BF_2_ unit of the BOPHY derivative, which affects the symmetry, sterics and planarity of the system, thereby enabling different packing possibilities and promoting pathway complexity. Initially, BOPHY 2 forms a kinetically controlled H-type supramolecular polymer (2A) in MCH that evolves over time into the thermodynamic product (2B) *via* a competitive pathway. According to the theoretical results, the BF_2_ groups build steric hindrance within the antiparallel H-type face-to-face stacks 2A, forcing the aggregates to rearrange into a more stable supramolecular polymer 2B with laterally displaced monomer units. This arrangement not only minimizes the steric hindrance but it also maintains the hydrogen bonds between the amide side groups, which were experimentally found to be stronger for the thermodynamic assembly (2B). Interestingly, this pathway complexity is absent in the case of the BODIPY derivative 1, since the H-type antiparallel dye arrangement is highly efficient due to the alternated, antiparallel orientation of the single, BF_2_ unit per monomer. We conclude that the main additional structural component of the BOPHY chromophore, *i.e*. the two BF_2_ groups, break the planarity of the π-system and enable different types of stacking interactions due to altered sterics and symmetry. This study highlights the versatility of the novel BOPHY chromophore in supramolecular self-assembly and introduces the break in planarity as a new molecular design strategy in controlled supramolecular polymerization.

## Data availability

All the experimental data have been shown in the ESI.†

## Author contributions

Rasitha Manha Veedu: synthesis, investigation, methodology, writing – original draft (lead). Zulema Fernández: theoretical calculations, writing – original draft (support). Nils Bäumer: AFM measurements, writing – review and editing. Antonia Albers: TEM measurements. Gustavo Fernández: conceptualization, writing – review and editing, supervision and funding acquisition.

## Conflicts of interest

There are no conflicts to declare.

## Supplementary Material

SC-015-D4SC02499K-s001

## References

[cit1] Ghosh S., Praveen V. K., Ajayaghosh A. (2016). Annu. Rev. Mater. Res..

[cit2] Babu S. S., Praveen V. K., Ajayaghosh A. (2014). Chem. Rev..

[cit3] Cao D., Liu Z., Verwilst P., Koo S., Jangjili P., Kim J. S., Lin W. (2019). Chem. Rev..

[cit4] S K., Sam B., George L., N S. Y., Varghese A. (2021). J. Fluoresc..

[cit5] Yang Q., Chang X., Lee J. Y., Olivera T. R., Saji M., Wisniewski H., Kim S., Zhang F. (2022). ACS Appl. Bio Mater..

[cit6] Tian J., Lin F., Yu S.-B., Yu J., Tang Q., Li Z.-T. (2022). Aggregate.

[cit7] Trenor S. R., Shultz A. R., Love B. J., Long T. E. (2004). Chem. Rev..

[cit8] Sakakibara K., Chithra P., Das B., Mori T., Akada M., Labuta J., Tsuruoka T., Maji S., Furumi S., Shrestha L. K., Hill J. P., Acharya S., Ariga K., Ajayaghosh A. (2014). J. Am. Chem. Soc..

[cit9] Li K., Duan X., Jiang Z., Ding D., Chen Y., Zhang G.-Q., Liu Z. (2021). Nat. Commun..

[cit10] Chen Z., Chen Z. (2023). Org. Chem. Front..

[cit11] Isobe A., Kajitani T., Yagai S. (2023). Angew. Chem., Int. Ed..

[cit12] Choi H., Ogi S., Ando N., Yamaguchi S. (2023). J. Am. Chem. Soc..

[cit13] Kim J.-K., Lee E., Kim M.-C., Sim E., Lee M. (2009). J. Am. Chem. Soc..

[cit14] Helmers I., Shen B., Kartha K. K., Albuquerque R. Q., Lee M., Fernández G. (2020). Angew. Chem., Int. Ed..

[cit15] Ogi S., Fukui T., Jue M. L., Takeuchi M., Sugiyasu K. (2014). Angew. Chem., Int. Ed..

[cit16] Ogi S., Stepanenko V., Thein J., Würthner F. (2016). J. Am. Chem. Soc..

[cit17] Chan M. H.-Y., Yam V. W.-W. (2022). J. Am. Chem. Soc..

[cit18] Sasaki N., Mabesoone M. F. J., Kikkawa J., Fukui T., Shioya N., Shimoaka T., Hasegawa T., Takagi H., Haruki R., Shimizu N., Adachi S., Meijer E. W., Takeuchi M., Sugiyasu K. (2020). Nat. Commun..

[cit19] Naranjo C., Adalid S., Gómez R., Sánchez L. (2023). Angew. Chem., Int. Ed..

[cit20] Martínez M. A., Doncel-Giménez A., Cerdá J., Calbo J., Rodríguez R., Aragó J., Crassous J., Ortí E., Sánchez L. (2021). J. Am. Chem. Soc..

[cit21] Han Y., Gao Z., Wang C., Zhong R., Wang F. (2020). Coord. Chem. Rev..

[cit22] Ghosh G. (2023). Giant.

[cit23] Lone M. U., Sahu N., Roy R. K., Adhikari B. (2023). Chem.–Eur. J..

[cit24] Wang F., Liao R., Wang F. (2023). Angew. Chem., Int. Ed..

[cit25] Kulkarni C., Meijer E. W., Palmans A. R. A. (2017). Acc. Chem. Res..

[cit26] Aida T., Meijer E. W. (2020). Isr. J. Chem..

[cit27] Bhosale S. V., Al Kobaisi M., Jadhav R. W., Morajkar P. P., Jones L. A., George S. (2021). Chem. Soc. Rev..

[cit28] Sarkar S., Laishram R., Deb D., George S. J. (2023). J. Am. Chem. Soc..

[cit29] Das G., Anand A., Vedhanarayanan B., Padmakumar A., Praveen V. K., Ajayaghosh A. (2023). Chem.–Eur. J..

[cit30] Khanra P., Singh A. K., Roy L., Das A. (2023). J. Am. Chem. Soc..

[cit31] Vázquez-González V., Mayoral M. J., Aparicio F., Martínez-Arjona P., González-Rodríguez D. (2021). ChemPlusChem.

[cit32] Wang Q., To W.-P., Chang X., Che C.-M. (2020). Chem.

[cit33] Barman S., Pal A., Mukherjee A., Paul S., Datta A., Ghosh S. (2024). Chem.–Eur. J..

[cit34] Yagai S., Kitamoto Y., Datta S., Adhikari B. (2019). Acc. Chem. Res..

[cit35] García F., Gómez R., Sánchez L. (2023). Chem. Soc. Rev..

[cit36] Wehner M., Würthner F. (2020). Nat. Rev. Chem.

[cit37] Matern J., Dorca Y., Sánchez L., Fernández G. (2019). Angew. Chem., Int. Ed..

[cit38] Ghosh G., Dey P., Ghosh S. (2020). Chem. Commun..

[cit39] Manha Veedu R., Niemeyer N., Bäumer N., Kartha Kalathil K., Neugebauer J., Fernández G. (2023). Angew. Chem., Int. Ed..

[cit40] Chen Z., Lohr A., Saha-Möller C. R., Würthner F. (2009). Chem. Soc. Rev..

[cit41] Fernández Z., Sánchez L., Babu S. S., Fernández G. (2024). Angew. Chem., Int. Ed..

[cit42] Grande V., Soberats B., Herbst S., Stepanenko V., Würthner F. (2018). Chem. Sci..

[cit43] Mayoral M. J., Guilleme J., Calbo J., Aragó J., Aparicio F., Ortí E., Torres T., González-Rodríguez D. (2020). J. Am. Chem. Soc..

[cit44] Rodríguez R., Naranjo C., Kumar A., Matozzo P., Das T. K., Zhu Q., Vanthuyne N., Gómez R., Naaman R., Sánchez L., Crassous J. (2022). J. Am. Chem. Soc..

[cit45] Cherumukkil S., Vedhanarayanan B., Das G., Praveen V. K., Ajayaghosh A. (2018). Bull. Chem. Soc. Jpn..

[cit46] Matarranz B., Fernández G. (2021). Chem. Phys. Rev..

[cit47] Zhang Y., Yuan S., Liu P., Jing L., Pan H., Ren X.-K., Chen Z. (2021). Org. Chem. Front..

[cit48] Ding J., Pan H., Wang H., Ren X.-K., Chen Z. (2022). Org. Chem. Front..

[cit49] Yang Y., Su X., Carroll C. N., Aprahamian I. (2012). Chem. Sci..

[cit50] Qian H., Cousins M. E., Horak E. H., Wakefield A., Liptak M. D., Aprahamian I. (2017). Nat. Chem..

[cit51] Araneda J. F., Piers W. E., Heyne B., Parvez M., McDonald R. (2011). Angew. Chem., Int. Ed..

[cit52] Patalag L. J., Jones P. G., Werz D. B. (2016). Angew. Chem., Int. Ed..

[cit53] Patalag L. J., Jones P. G., Werz D. B. (2017). Chem.–Eur. J..

[cit54] Tamgho I.-S., Hasheminasab A., Engle J. T., Nemykin V. N., Ziegler C. J. (2014). J. Am. Chem. Soc..

[cit55] Rödle A., Ritschel B., Mück-Lichtenfeld C., Stepanenko V., Fernández G. (2016). Chem.–Eur. J..

[cit56] Dorca Y., Naranjo C., Ghosh G., Soberats B., Calbo J., Ortí E., Fernández G., Sánchez L. (2021). Chem. Sci..

[cit57] Jiang L., Gao H., Gai L., Shen Z. (2018). New J. Chem..

[cit58] Yamagata H., Maxwell D. S., Fan J., Kittilstved K. R., Briseno A. L., Barnes M. D., Spano F. C. (2014). J. Phys. Chem. C.

[cit59] Hestand N. J., Spano F. C. (2017). Acc. Chem. Res..

[cit60] Chakraborty S., Debnath P., Dey D., Bhattacharjee D., Hussain S. A. (2014). J. Photochem. Photobiol., A.

[cit61] Helmers I., Niehues M., Kartha K. K., Ravoo B. J., Fernández G. (2020). Chem. Commun..

[cit62] Eikelder H. M. M., Markvoort A. J., de Greef T. F. A., Hilbers P. A. J. (2012). J. Phys. Chem. B.

[cit63] Korevaar P. A., Schaefer C., de Greef T. F. A., Meijer E. W. (2012). J. Am. Chem. Soc..

[cit64] Wehner M., Röhr M. I. S., Bühler M., Stepanenko V., Wagner W., Würthner F. (2019). J. Am. Chem. Soc..

[cit65] Rubert L., Islam M. F., Greytak A. B., Prakash R., Smith M. D., Gomila R. M., Frontera A., Shimizu L. S., Soberats B. (2023). Angew. Chem., Int. Ed..

[cit66] Bujosa S., Doncel-Giménez A., Bäumer N., Fernández G., Ortí E., Costa A., Rotger C., Aragó J., Soberats B. (2022). Angew. Chem., Int. Ed..

[cit67] Bäumer N., Castellanos E., Soberats B., Fernández G. (2023). Nat. Commun..

[cit68] Matern J., Bäumer N., Fernández G. (2021). J. Am. Chem. Soc..

[cit69] Kulkarni C., Korevaar P. A., Bejagam K. K., Palmans A. R. A., Meijer E. W., George S. J. (2017). J. Am. Chem. Soc..

[cit70] Ghosh G., Chakraborty A., Pal P., Jana B., Ghosh S. (2022). Chem.–Eur. J..

[cit71] Langenstroer A., Kartha K. K., Dorca Y., Droste J., Stepanenko V., Albuquerque R. Q., Hansen M. R., Sánchez L., Fernández G. (2019). J. Am. Chem. Soc..

[cit72] Becke A. D. (1993). J. Chem. Phys..

[cit73] Francl M. M., Pietro W. J., Hehre W. J., Binkley J. S., Gordon M. S., DeFrees D. J., Pople J. A. (1982). J. Chem. Phys..

