# Supplementary material for: Pathway-dependent supramolecular polymerization by planarity breaking
Source: Chem Sci. 2024 Jun 11;15(28):10745–52. doi: 10.1039/d4sc02499k (PMC11253169; doi:10.1039/d4sc02499k)
Supplement: SC-015-D4SC02499K-s001 [file SC-015-D4SC02499K-s001.pdf]

Electronic Supplementary Material (ESI) for Chemical Science.
This journal is © The Royal Society of Chemistry 2024

Pathway-dependent supramolecular polymerization by planarity breaking

Rasitha Manha Veedu,[a] Zulema Fernández,[a] Nils Bäumer,[a] Antonia Albers,[a] Gustavo Fernández*[a]

[a] Universität Münster, Organisch-Chemisches Institut, Corrensstraße 36, 48149 Münster, Germany. e-mail: fernandg@uni-muenster.de

Table of contents

Experimental procedures......3

Materials and Methods......3

Synthetic procedures and Characterization......4

Results and Discussion......6

1. **Nucleation-elongation model for supramolecular polymerization**......6
2. **Denaturation model for supramolecular polymerization**......6
3. **Thermodynamic parameters**......7
4. **Fluorescence quantum yield**......8
5. **Supplementary figures**......9
6. **Morphological studies**......17
7. **Theoretical calculations**......21

References......25

Experimental Procedures

Materials and Methods

Chemicals and Reagents: All chemicals were purchased from Sigma Aldrich (St. Louis, MO, USA), TCI Europe N.V. (Tokyo, JP) and BLD pharm (Senefelder ring, Reinbeck, DE) and used without further purification methods unless otherwise mentioned. Silica gel was used for column chromatography unless otherwise mentioned.

Column chromatography: Preparative column chromatography was performed in self-packed glass columns of different sizes with silica gel (particle size: 40-60 µm, *Merck*). Solvents were distilled before usage.

NMR spectroscopy: ^{1}H and ^{13}C NMR spectra were recorded at 298 K on Avance II 300 and Avance II 400 from Bruker for routine experiments using tetramethylsilane (TMS) as internal standard. Additional ^{1}H as well as 2D ^{1}H-^{19}F HOESY spectra were recorded on an Agilent DD2 500 (^{1}H: 500 MHz) and an Agilent DD2 600 (^{1}H: 600 MHz) at a standard temperature of 298 K in deuterated solvents. Multiplicities for proton signals are abbreviated as s, d, t, q and m for singlet, doublet, triplet, quadruplet and multiplet, respectively.

Mass spectrometry (MS): MALDI mass spectra were recorded on a Bruker Daltonics Ultraflex ToF/ToF or a Bruker Daltonics Autoflex Speed with a SmartBeamTM NdYAF-Laser with a wavelength of 335 nm. ESI mass spectra were measured on a Bruker MicrOToF system. The signals are described by their mass/charge ratio (m/z) in u.

UV-Vis spectroscopy: UV-Vis absorption spectra were recorded on a JASCO V-770 or a JASCO V-750 with a spectral bandwidth of 1.0 nm and a scan rate of 400 nm min^{-1}. Glass cuvettes with an optical length of 1 cm, 1 mm and 0.1 mm were used. All measurements were conducted in commercially available solvents of spectroscopic grade.

Fluorescence spectroscopy: Fluorescence and excitation spectra were recorded on a JASCO Spectrofluorometer FP-8500 in quartz cuvettes (SUPRASIL®, Hellma) with an optical length of 1 cm.

FT-IR spectroscopy: Solution and solid-state measurements were carried out using a JASCO-FT-IR-6800 equipped with a CaF_2 cell with a path length of 0.1 mm.

Atomic force microscopy (AFM): The AFM images were recorded on a Multimode®8 SPM System manufactured by Bruker AXS. The used cantilevers were AC200TS by Oxford Instruments with an average spring constant of 9 N m^{-1}, an average frequency of 150 kHz, an average length of 200 µm, an average width of 40 µm and an average tip radius of 7 nm. All samples were drop-casted from freshly prepared solutions onto an HOPG surface.

Gel permeation chromatography (GPC): Gel permeation chromatography was performed on a Shimadzu prominence GPC system equipped with two Tosoh TSKgel columns (G2500H XL; 7.8 mm I.D. x 30 cm, 5 µm; Part. No. 0016135) using CH_2Cl_2 as eluent. The solvent flow was set to be 1 mL/min. Detection was carried out *via* a Shimadzu prominence SPD-M20A diode array detector (DAD).

Scanning electron microscopy (SEM): SEM images of self-assembled species were recorded on a Thermo Fisher Scientific Phenom ProX Desktop SEM. All samples were drop-casted on a silicon wafer surface.

Transmission electron microscopy (TEM): The TEM images were recorded on a FEI TITAN Themis G3 60-300 transmission electron microscope manufactured by Thermo Fischer Scientific with an operation voltage of 60 kV and 300 kV. The X-FEG field emission gun gives a bright and highly stable electron source for the measurements for high resolution images. This device is also equipped with monochromator, Cs image corrector, quadruple EDX-system, Fischione model 3000 HAADF detector, a fast CMOS camera to capture high resolution images with very fast frame rates and a high-resolution EEL spectrometer (GATAN Quantum 965) for detailed analysis of the structures. The samples were prepared on carbon coated mesh copper grid by drop casting the sample and the excess liquid was drained using a filter paper that was placed under the grid.

Synthetic procedures and characterization

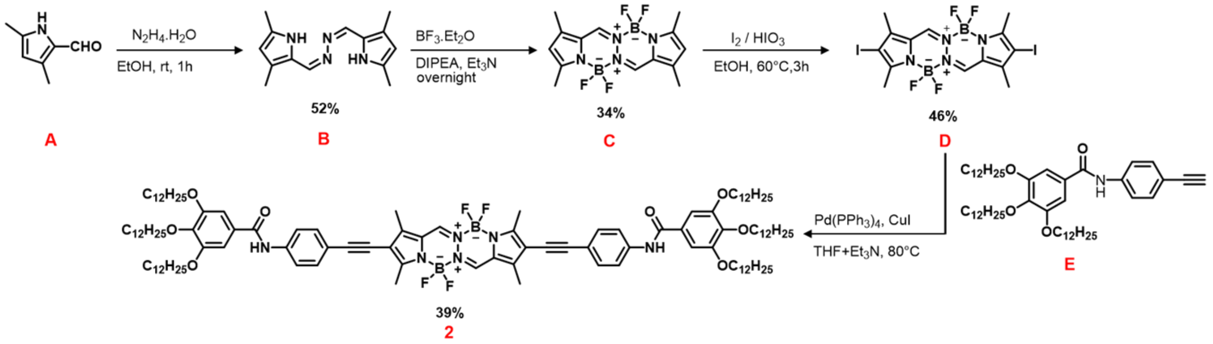

Figure S1: Synthesis route to obtain final compound **2**.

Compound **1**, **B**, **C**, **D** and 3,4,5-tris(dodecyloxy)-N-(4-ethynylphenyl)benzamide (**E**) were prepared by following the reported synthetic procedures and showed similar spectroscopic properties to those reported therein. [1,2]

Synthesis of linear BOPHY derivative (2):

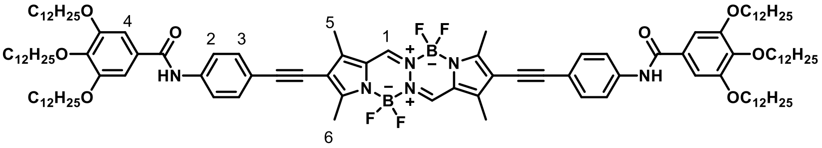

3,4,5-tris(dodecyloxy)-N-(4-ethynylphenyl)benzamide (**E**) (0.4g, 0.516 mmol, 2.2 eq), diiodo bophy derivative (**D**) (0.138g, 0.234 mmol, 1 eq), tetrakis(triphenylphosphine)palladium(0) (27 mg, 0.0234 mmol, 10 mol%) and copper(I) iodide (2.2 mg, 0.0117 mmol, 5 mol%) were dissolved in a 4:1 mixture of degassed THF: Et_3N (5 mL). The solution was stirred at 80°C for 12 hours. The solvent was removed under reduced pressure and the crude product was purified by column chromatography running an increasing toluene:ethyl acetate gradient (10:1). The product was further purified by several runs in a gel permeation chromatography using $CHCl_3$ as eluent yielding the pure product as an orange solid with 39% yield.

^{1}H NMR ($CDCl_3$, 500 MHz) δ (ppm) = 7.98 (s, 2H, NH), 7.81 (s, 2H, H_1), 7.66 (d, 4H, H_2), 7.53 (d, 4H, H_3), 7.05 (s, 4H, H_4), 4.02 (m, 12H, OCH_2), 2.63 (s, 6H, H_5), 2.45 (s, 6H, H_6), 1.85-1.71 (m,12H), 1.54-1.44 (m, 12H), 1.39-1.23 (m, 96H), 0.88 (t, 9H, OCH_3).

^{13}C NMR ($CDCl_3$, 100 MHz) δ (ppm) = 165.71, 153.76, 153.45, 142.15, 141.94, 138.29, 135.34, 133.61, 132.45, 129.72, 122.59, 119.94, 119.01, 114.30, 106.04, 95.82, 80.49, 73.75, 69.68, 32.08, 30.49, 29.91, 29.52, 26.24, 22.84, 14.26, 13.38, 10.77.

ESI-MS (TOF): m/z 1883.39402 [M+H] calculated for $C_{116}H_{179}B_2F_4N_6O_8$: 1883.39527

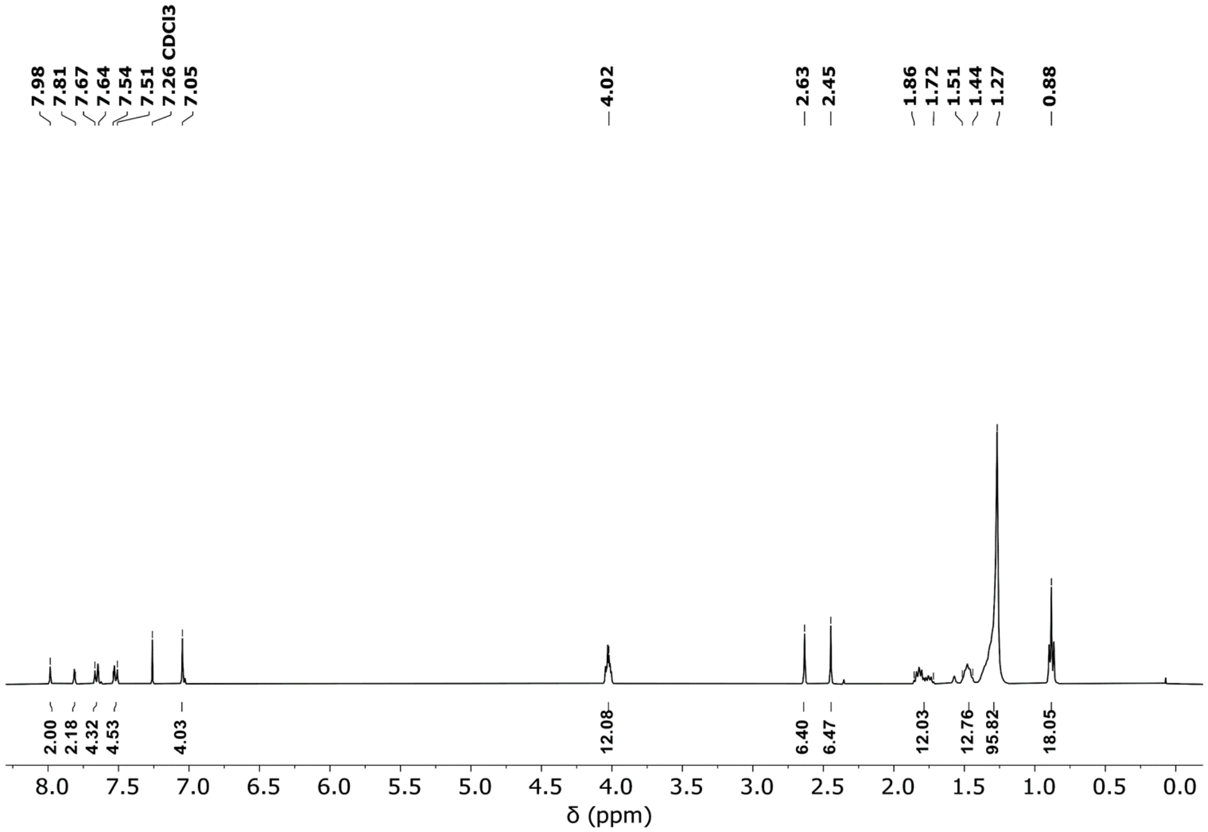

Figure S2: ^{1}H NMR ($CDCl_3$, 500 MHz) spectrum of compound **2**.

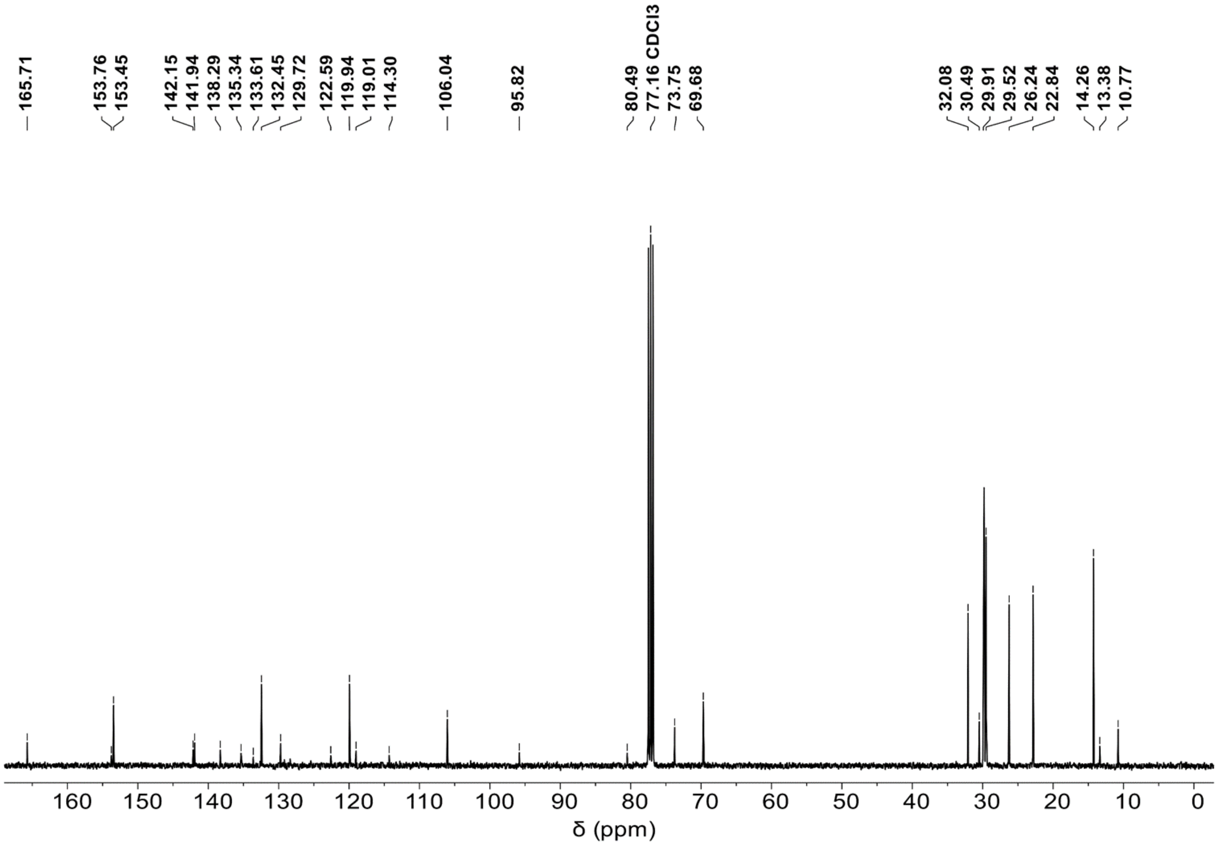

Figure S3: ^{13}C NMR ($CDCl_3$, 100 MHz) spectrum of compound **2**.

Results and Discussion

1. Nucleation-Elongation model for cooperative supramolecular polymerization

The equilibrium between the monomeric and supramolecular polymer species can be described in a cooperative process with the Nucleation-Elongation model which was developed by Ten Eikelder, Markvoort and Meijer.[3, 4] This model is used to describe the aggregation of **2**, which exhibits a non-sigmoidal cooling curve as shown in temperature-dependent UV-Vis experiments. The model extends nucleation-elongation based equilibrium models for growth of supramolecular homopolymers to the case of two monomer and aggregate types and can be applied to symmetric supramolecular copolymerizations, as well as to the more general case of nonsymmetric supramolecular copolymerizations. In a cooperative process, the polymerization occurs via two steps: in a first step (nucleation), a nucleus, which is assumed to have a size of 2 molecules, is formed. In a subsequent step, the elongation of the nuclei into one-dimensional supramolecular polymers occurs. The values T_e, $\Delta H°_{nucl}$, $\Delta H°$ and $\Delta S°$ can be determined by a non-linear least-square analysis of the experimental melting curves. The equilibrium constants associated with the nucleation and elongation phases can be calculated using the following equations:

Nucleation step: $K_n = e\left(\frac{-\left(\left(\Delta H^0 - \Delta^0_{H_{NP}}\right) - T\Delta S^0\right)}{RT}\right)$ *(1)*

Elongation step: $K_e = e\frac{(-(\Delta H^0 - T\Delta S^0))}{RT}$ *(2)*

And the cooperative factor (σ) is given by:

$\sigma = \frac{k_n}{k_e} = e\left(\frac{\Delta H_{NP}}{RT}\right)$ *(3)*

2. Denaturation Model for Supramolecular Polymerization

The denaturation model[5] is based on the concentration-dependent supramolecular polymerization equilibrium model by Goldstein,[6] where the polymerization is described as a sequence of monomer addition equilibria.

$[P_n] = K_n\,[P_n - 1]\,[X]$ *(4)*

$[P_{n+1}] = K_e\,[P_n][X]$ *(5)*

$[P_i] = K_e\,[P_i - 1]\,[X]$ *(6)*

For the cooperative model, $K_n < K_e$ and for the isodesmic process $K_n = K_e$. The concentration for each species P_i is given by $[P_i] = K^{i-1}{}_n\,[X]^i$ for $i \leq n$ and $[P_i] = K^{i-n}{}_e\,K^{n-1}{}_n\,[X]^i$ for $i > n$.

The dimensionless mass balance is obtained by inserting the dimensionless concentration $p_i = k_e[P_i]$, the monomer concentration x= K_e [X] and the concentration of each species P_i (for i≤ n): $p_i = \sigma^{i-1}x^i$ and for i > n : $p_i = \sigma^{n-1}x^i$):

$$x_{tOt} = \sigma^{-1}\sum_{i=n}^{n} i(\sigma x)^i + \sigma^{n-1}\Sigma_{i=n+1}^{\infty} i x^i \qquad (7)$$

Both sums are evaluated by using standard expressions for converging series:

$$x_{tot} = \left(\frac{(\sigma x)^{n+1}(n\sigma x - n - 1)}{(\sigma x - 1)^2} + \frac{\sigma x}{(\sigma x - 1)^2}\right) - \sigma^{n-1}\left(\frac{x^{n+1}(n\varkappa - n - 1)}{[x-1]^2}\right) \qquad (8)$$

With $x_{tot} = C_{tot}k_e$ and c_{tot} = total monomer concentration

The sum solved by standard numerical methods (Matlabfzerosolver) yields the dimensionless monomer concentration x. Considering that every species with is defined as aggregate, the degree of i > 1 aggregation results in:

$$\alpha_{agg} = \frac{x_{tot} - x}{x_{tot}} \qquad (9)$$

Via $k_e = e^{\left(-\frac{\Delta G^0}{RT}\right)}$ the denaturation curves can be obtained with *f* defined as volume fraction of good solvent:

$$\Delta G^0 = \Delta G^0 + mf \qquad (10)$$

It is assumed that the cooperativity factor σ is independent of the volume fraction and the m value for the elongation regime equals the m value for nucleation. The denaturation data needs to be transformed into the normalized degree of aggregation, if fitted to the supramolecular polymerization equilibrium model:

$$(f) = \frac{A(f)-A(f=0)}{A(f=1)-A(f=0)} \qquad (11)$$

The optimization of the four needed parameters (ΔG_0, m, σ and p) to fit the equilibrium model to the experimental data (normalized degree *vs*. *f*) is done by the non-linear least-squares analysis using Matlab (lsqnonlinsolver). The data is then fitted with the non-linear least squared regression (Levenberg Marquardt algorithm).

3. Thermodynamic parameters

The thermodynamic parameters (Table S1 and Table S2) of **2A** and **2B** were calculated by fitting the respective experimental data to the nucleation-elongation model[3,4] and denaturation model.[6] Variable temperature fits were determined by simultaneously fitting multiple concentrations (**2A**: 5, 7, 10, 15 µM MCH; **2B**: 15, 20,30 µM MCH for VT UV-Vis measurements, **2A** & **2B**: 10, 20, 30 µM MCH for denaturation experiments).

Table S1: Thermodynamic parameters obtained for **2A** (505 nm) derived from VT UV-Vis studies in MCH: [3,4]

c (µM)	ΔH_0 (kJ/mol)	ΔH_0(STD) (kJ/mol)	ΔS_0 (kJ/mol)	ΔS_0(STD) (kJ/mol)	ΔH_{Nucl} (kJ/mol)	ΔH_{Nucl}(STD) (kJ/mol)	T_e (K)	N	ΔG_0 (kJ/mol)	K_{el}	K_{nucl}	σ
5	-94.91	0.7046	-0.2012	0.0023	-12.689	0.1862	313.4	199958	-34.90	200014	0.0076	0.00768
7	-88.11	1.0156	-0.1809	0.0023	-12.116	0.2618	315.1	142414	-34.18	142860	0.0098	0.00982
10	-113.91	3.0509	-0.2631	0.0098	-11.015	0.5710	317.4	99099.2	-35.46	100003	0.0154	0.01540
15	-102.23	1.1892	-0.2245	0.0038	-11.475	0.2584	322.6	66449.5	-35.29	66667	925.05	0.01388
**ϕ = ΔG_0 = -35.20 KJ/mol**												

Table S2: Thermodynamic parameters obtained for **2B** (600nm) derived from VT UV-Vis studies in MCH:[3,4]

c (µM)	ΔH_0 (kJ/mol)	ΔH_0(STD) (kJ/mol)	ΔS_0 (kJ/mol)	ΔS_0(STD) (kJ/mol)	ΔH_{Nucl} (kJ/mol)	ΔH_{Nucl}(STD) (kJ/mol)	T_e (K)	N	ΔG_0 (kJ/mol)	K_{el}	K_{nucl}	σ
15	-94.52	3.666313	-0.1883	0.01110	-21.632	2.69672	335.01	67298	-38.37	66668	29.40	4.41×10^{-4}
20	-171.80	1.448422	-0.4246	0.00439	-21.359	0.73743	342.67	49975.3	-45.20	9850.65	5.489	5.57×10^{-4}
30	-144.14	4.047898	-0.3363	0.01210	-13.227	0.73950	345.61	33824.5	-43.84	15975.46	160.4	0.00104
**ϕ = ΔG_0 = -42.47 KJ/mol**												

Table S3: Thermodynamic parameters obtained for **2A** & **2B** derived from denaturation experiments in MCH/$CHCl_3$ mixture at room temperature:[6]

	**2A** (505nm) ΔG_0 (kJ/mol)	**2B** (575nm) ΔG_0 (kJ/mol)
10	-36.2	-40.05
20	-34.4	-40.90
30	-35.6	-41.10
Φ	**-35.4**	**-40.68**

4. Fluorescence quantum yield

Absolute luminescence quantum yields were measured on a JASCO spectrofluorometer FP-8500 (equipped with an ILF-835 integrating sphere) with a band width of 5 nm and a scan rate of 1000 nm/min. Quartz cuvettes with an optical path of 5 mm were employed. The measurements were carried out with a specific excitation wavelength for each sample, as shown in Table S4.

Table S4: Fluorescence quantum yields of monomer, **2A** and **2B**.

	Solvent	ϕ_F
Monomer	$CHCl_3$ (λ_{ex}= 495 nm)	97.3%
**2A**	MCH (λ_{ex} = 454 nm)	87.3%
**2B**	MCH (λ_{ex} = 474 nm)	12.7%

5. Supplementary Figures

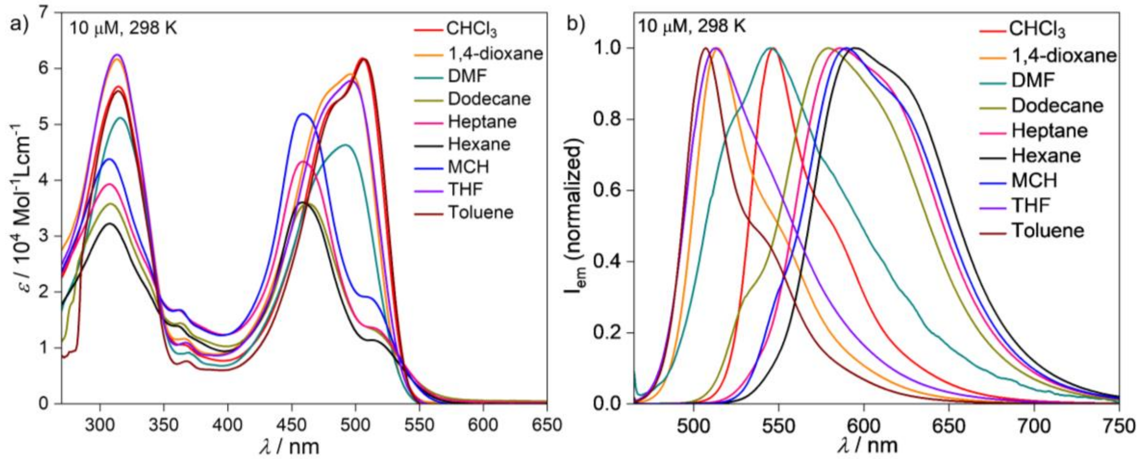

Figure S4: Solvent-dependent absorption (a) and normalized emission spectra (b) of compound **2** at 298 K (c = 10 μM).

Minor shifts in emission in solvents such as toluene arise from solvatochromism, which takes place without any aggregation process.

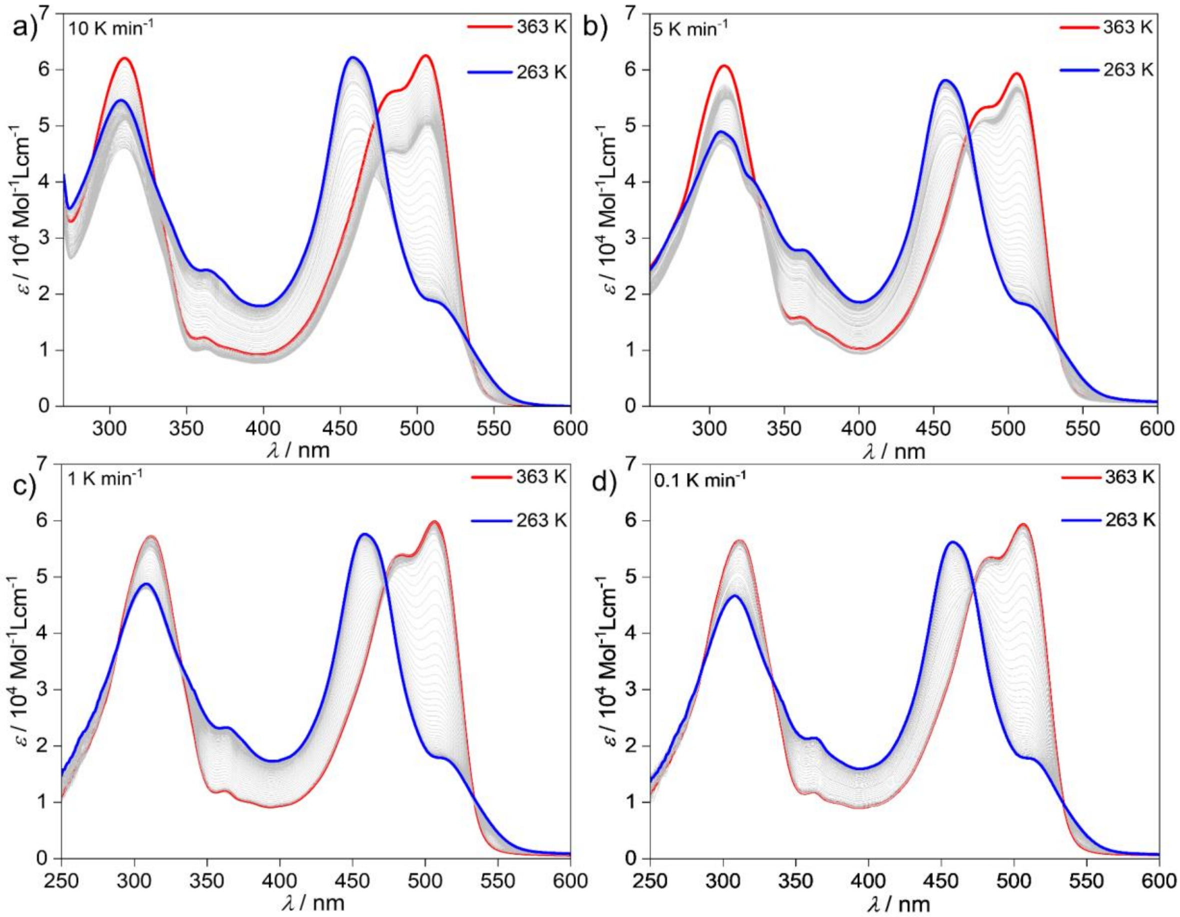

Figure S5: Variable Temperature (VT) UV-Vis spectra obtained upon cooling solutions of **2** at different cooling rates: a) 10 K min^{-1} b) 5 K min^{-1} c) 1 K min^{-1} and d) 0.1 K min^{-1} (c = 10 μM in MCH).

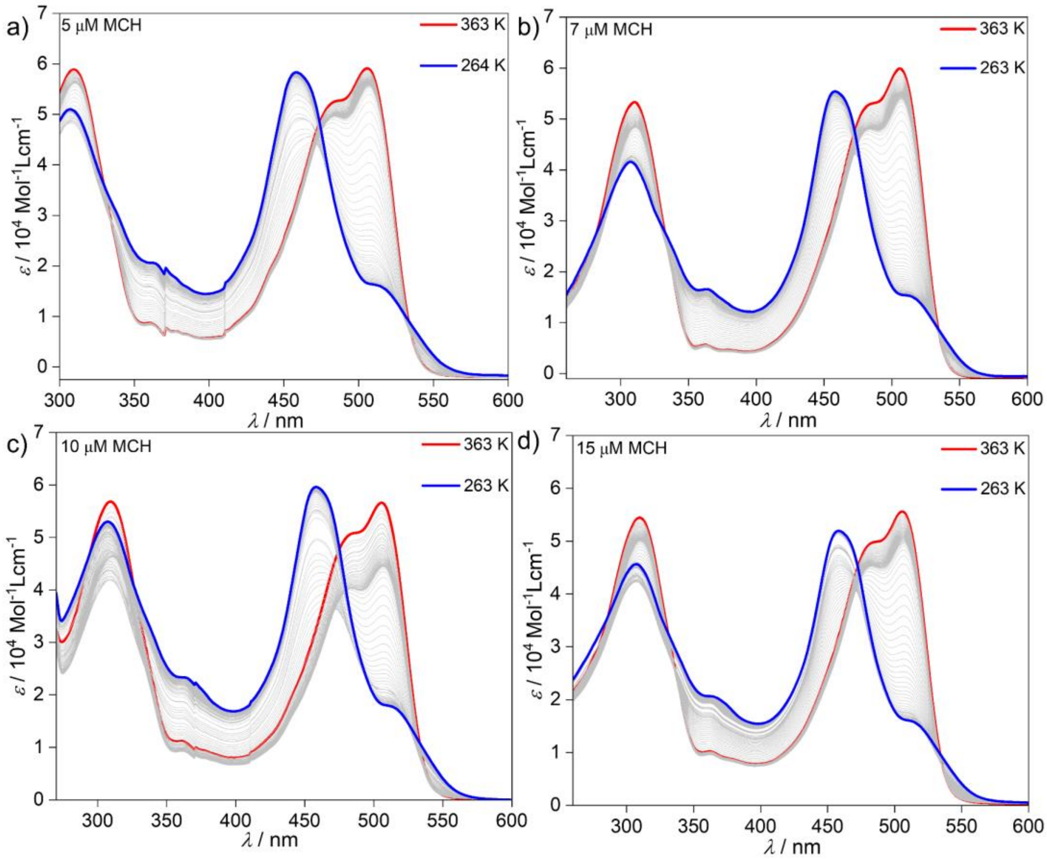

Figure S6: Variable Temperature (VT) UV-Vis spectra obtained upon cooling solutions of **2** at different concentrations: a) 5 µM b) 7 µM c) 10 µM and d) 15 µM with a cooling rate of 1 K min^{-1} in MCH.

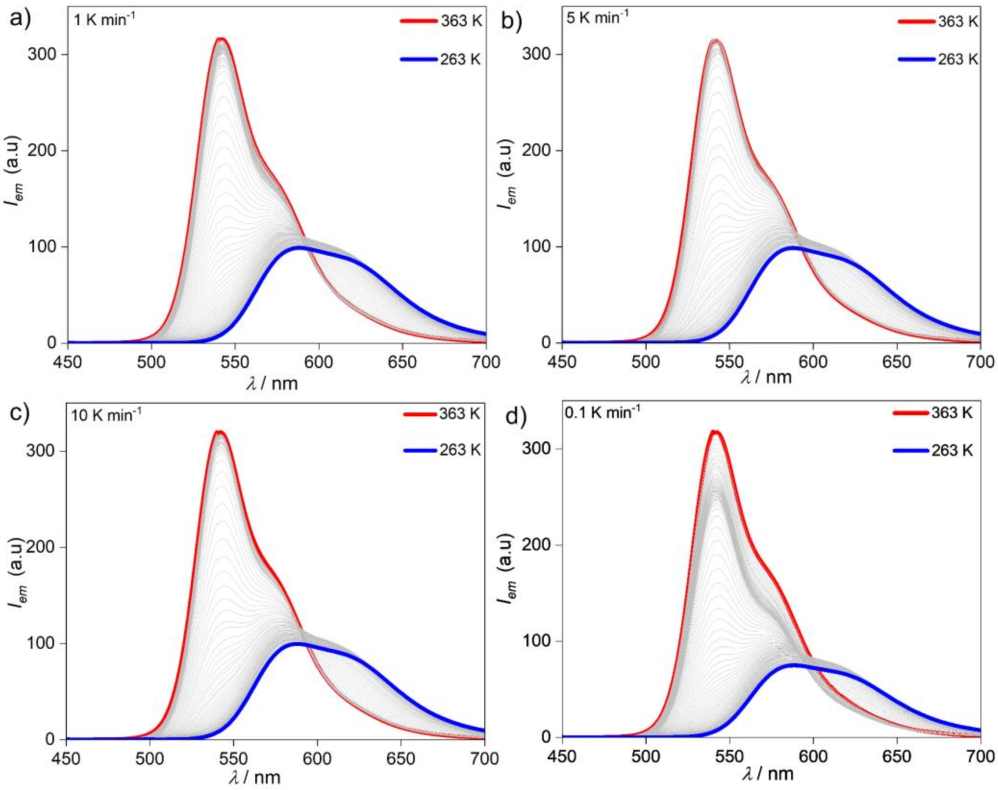

Figure S7: Variable Temperature (VT) emission spectra obtained upon cooling solutions of **2** at different cooling rates: a) 10 K min^{-1} b) 5 K min^{-1} c) 1 K min^{-1} and d) 0.1 K min^{-1} (c = 10 µM in MCH). λ_{ex} = 440 nm.

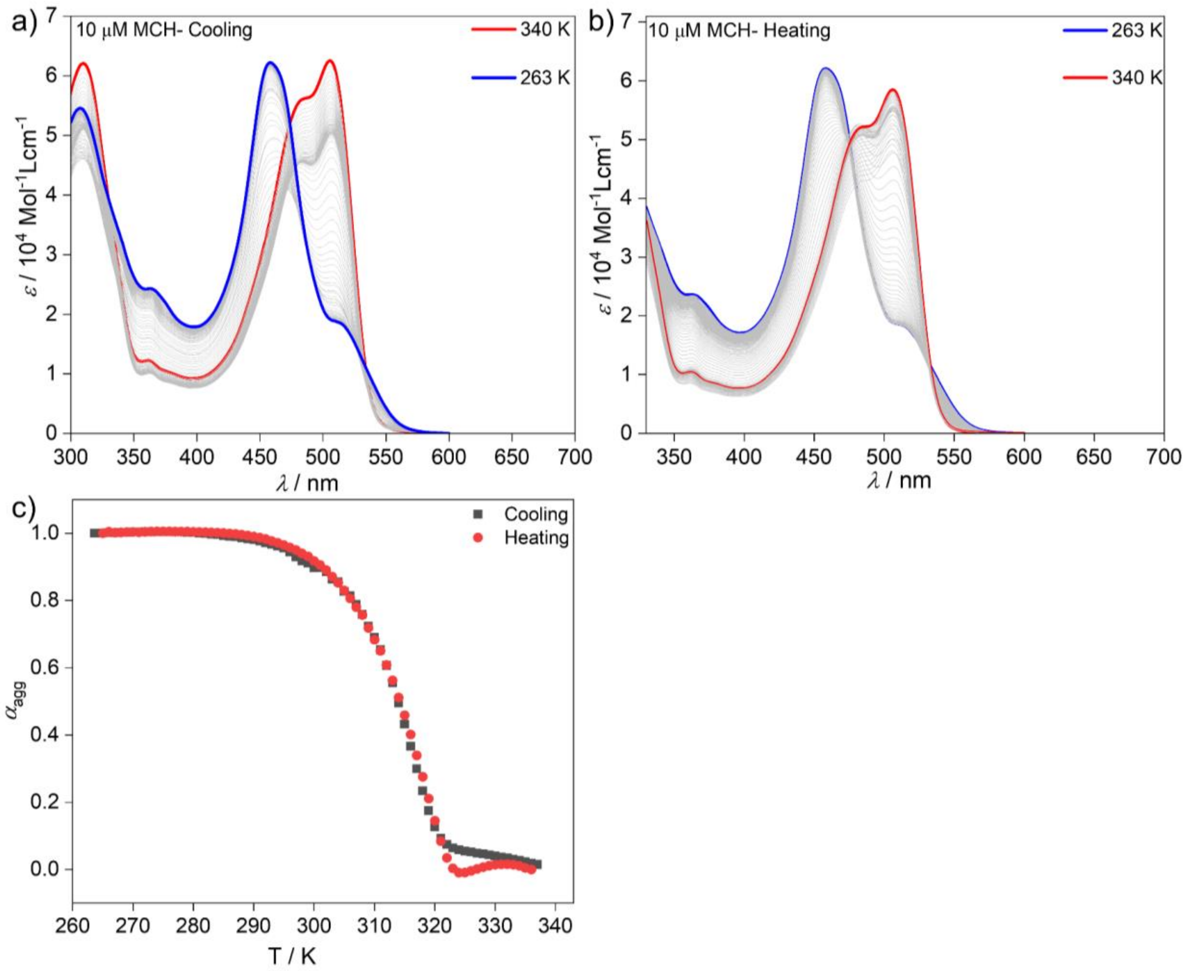

Figure S8: Variable Temperature (VT) cooling (a) and heating (b) UV-Vis spectra of a 10 µM MCH solution of **2** with a cooling/heating rate of 1 K min^{-1}. c) α_{agg} *vs. T* at wavelength of 505 nm.

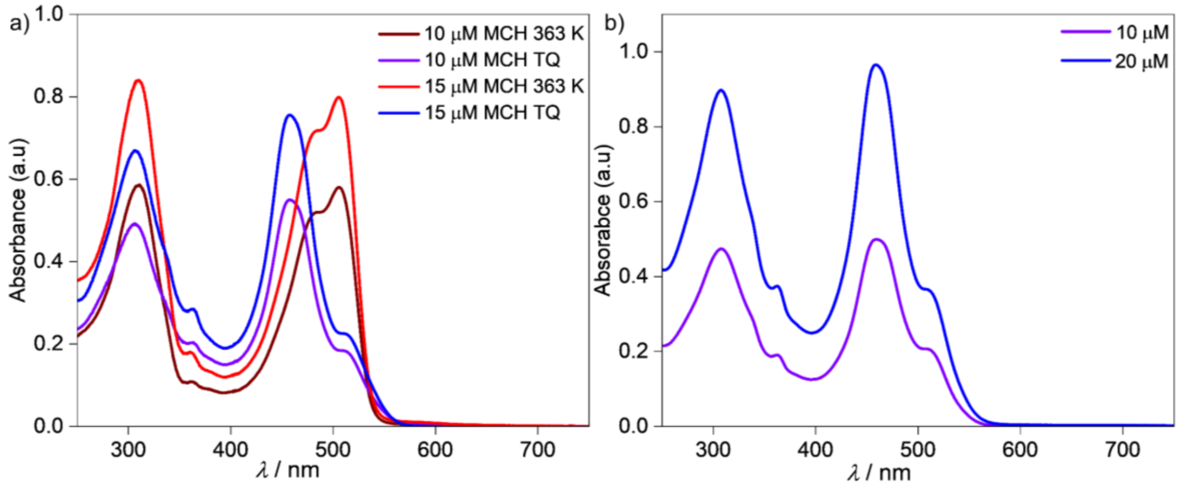

Figure S9: UV-Vis spectra of compound **2** prepared upon: a) thermal quenching (TQ); b) solvophobic quenching.

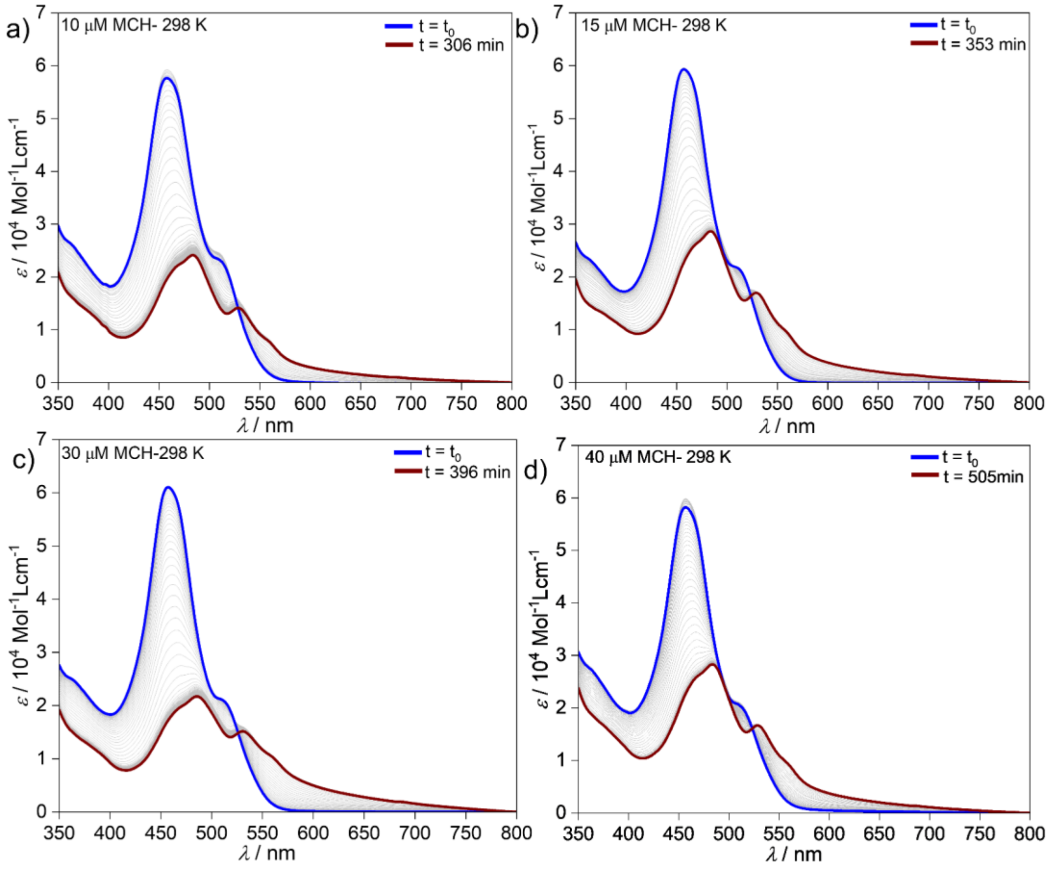

Figure S10: Time-dependent evolution of **2A** into **2B** of compound **2** at different concentrations: a) 10 μM b) 15 μM c) 30 μM and d) 40 μM at 298 K in MCH.

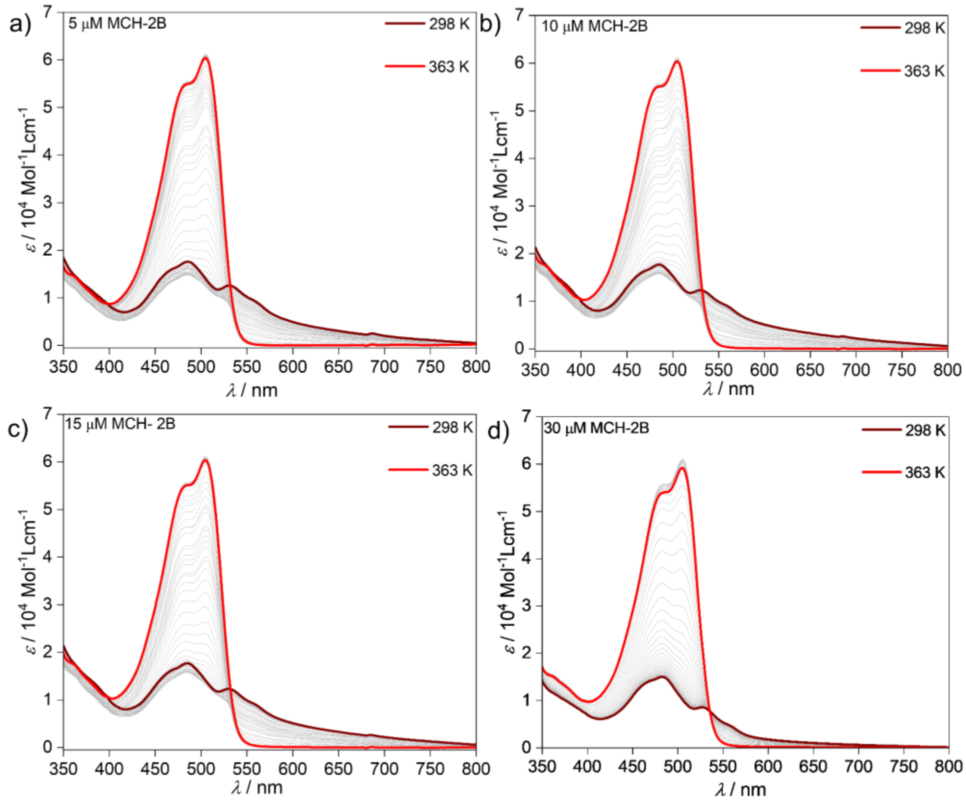

Figure S11: Variable Temperature (VT) UV-Vis spectra obtained upon heating a sonicated solution of **2A** of compound **2** at different concentrations: a) 5 µM b) 10 µM c) 15 µM and d) 30 µM with a heating rate of 1 K min^{-1} in MCH.

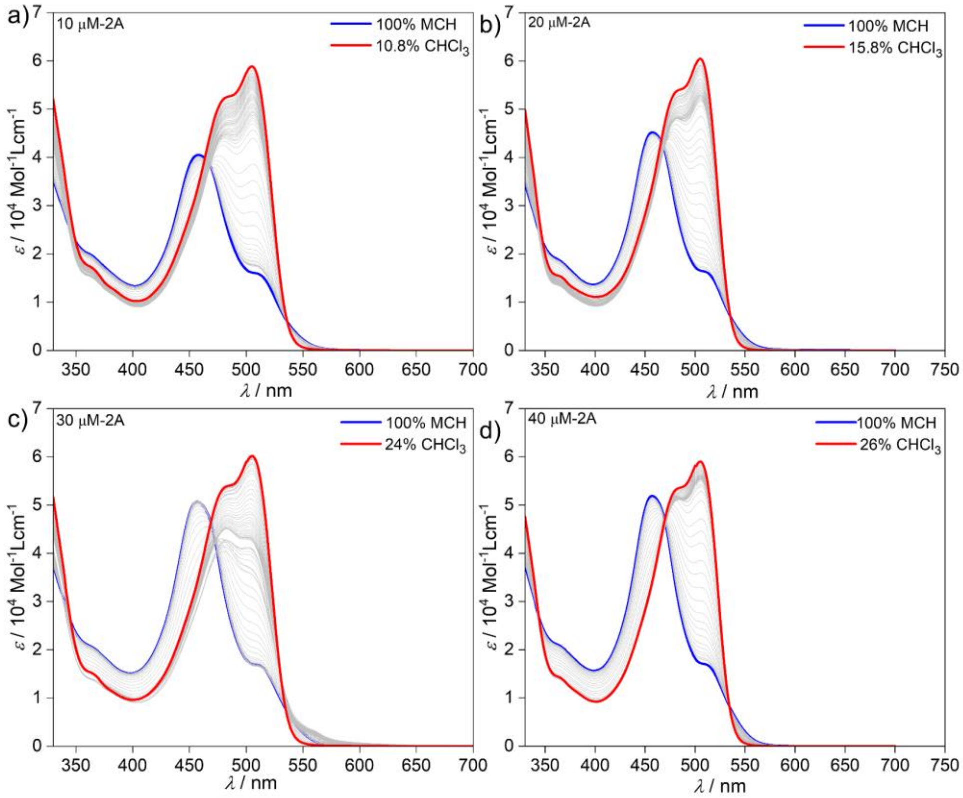

Figure S12: Solvent-dependent UV-Vis studies of **2A** upon monitoring the disassembly *via* an increasing volume fraction of $CHCl_3$ at constant concentration: a) 10 µM b) 20 µM c) 30 µM and d) 40 µM at 298 K.

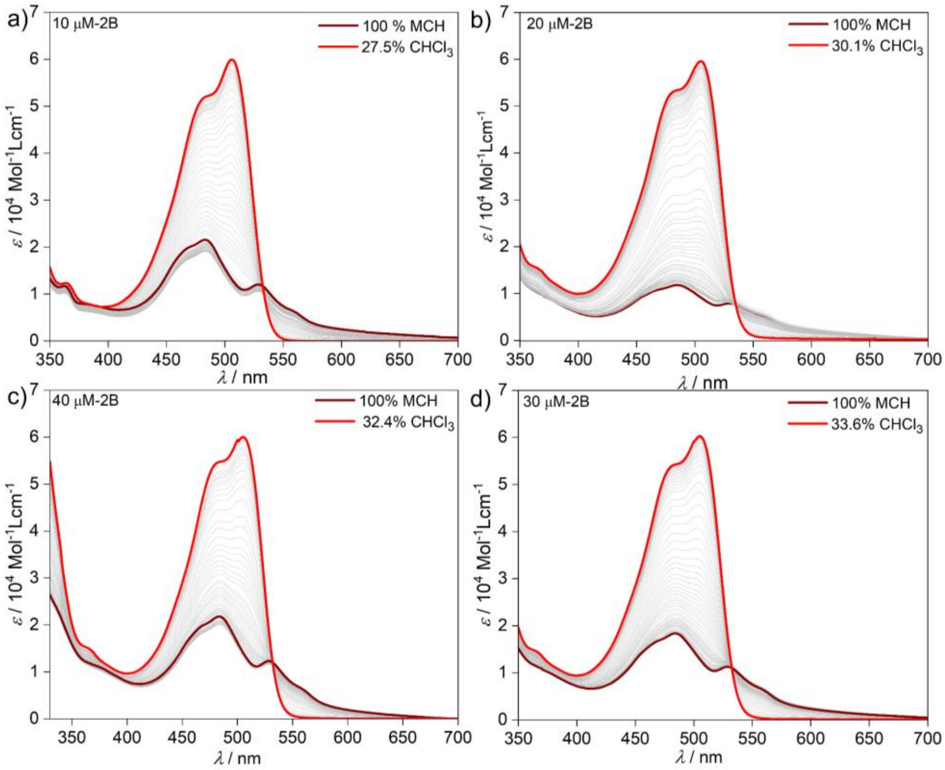

Figure S13: Solvent-dependent UV-Vis studies of **2B** upon monitoring the disassembly *via* an increasing volume fraction of $CHCl_3$ at constant concentration: a) 10 µM b) 20 µM c) 30 µM and d) 40 µM at 298 K.

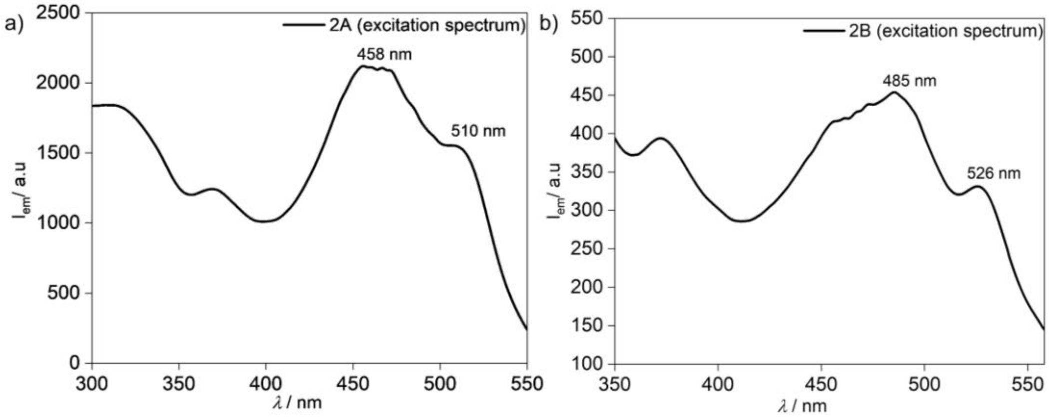

Figure S14: Excitation spectra of aggregate a) **2A** (λ_{em}= 570 nm) and b) **2B** (λ_{em}= 570 nm).

Excitation spectra of aggregate **2A** (λ_{em}= 570 nm) and **2B** (λ_{em}= 570 nm) reproduced their corresponding absorption features. The absence of any significant spectral shift or broadening in the excitation spectra of aggregate **2A** and **2B** together with the lack of a monomeric pattern rules out excimer formation in the aggregated species.

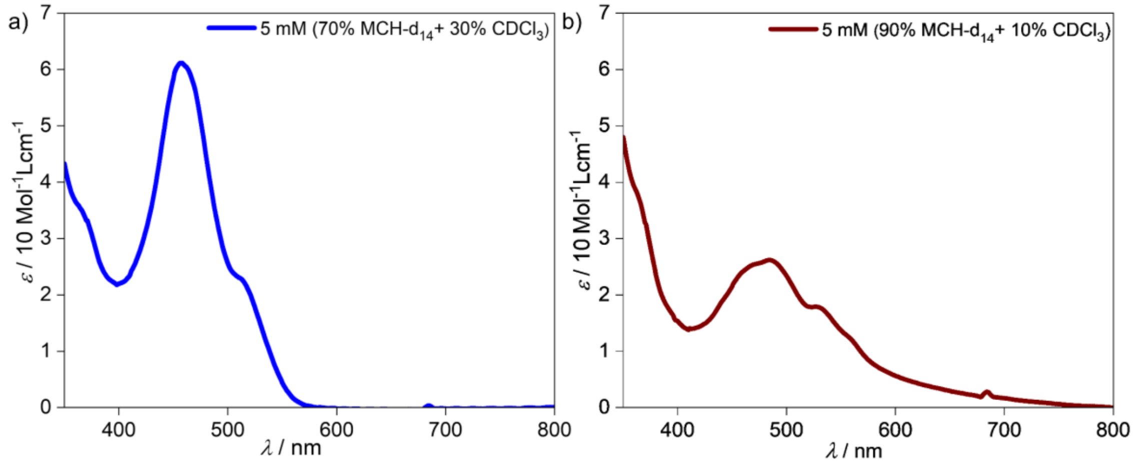

Figure S15: UV-Vis spectra of: a) **2A** (70% MCH-d_{14}+30% $CDCl_3$); b) **2B** (90% MCH-d_{14}+10% $CDCl_3$) at a concentration of $c = 5 \times 10^{-3}$ M and at 328 K.

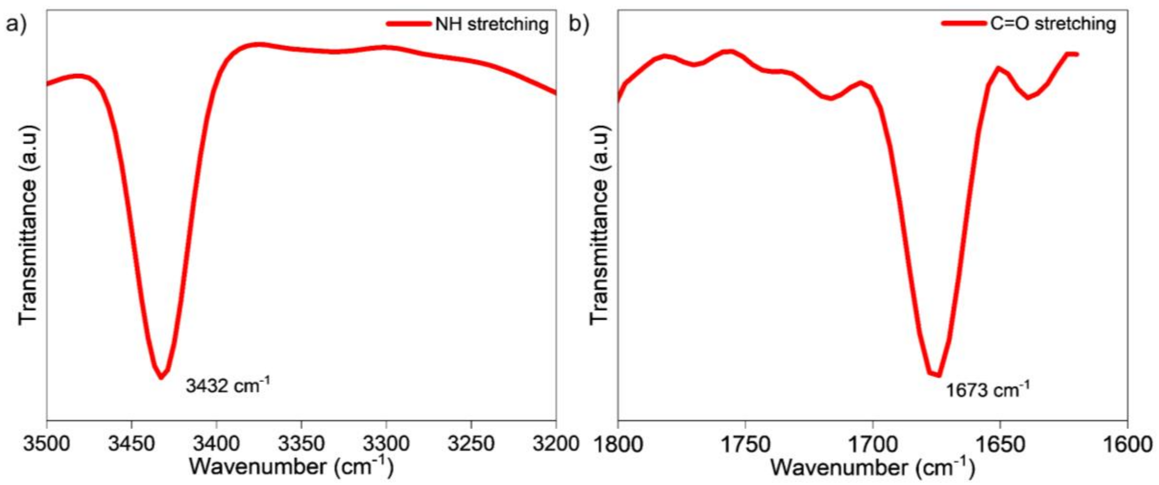

Figure S16: Partial FT-IR spectrum of the monomer ($CHCl_3$, $c = 1 \times 10^{-3}$ M) showing regions of a) N-H and b) carbonyl C=O stretching frequencies.

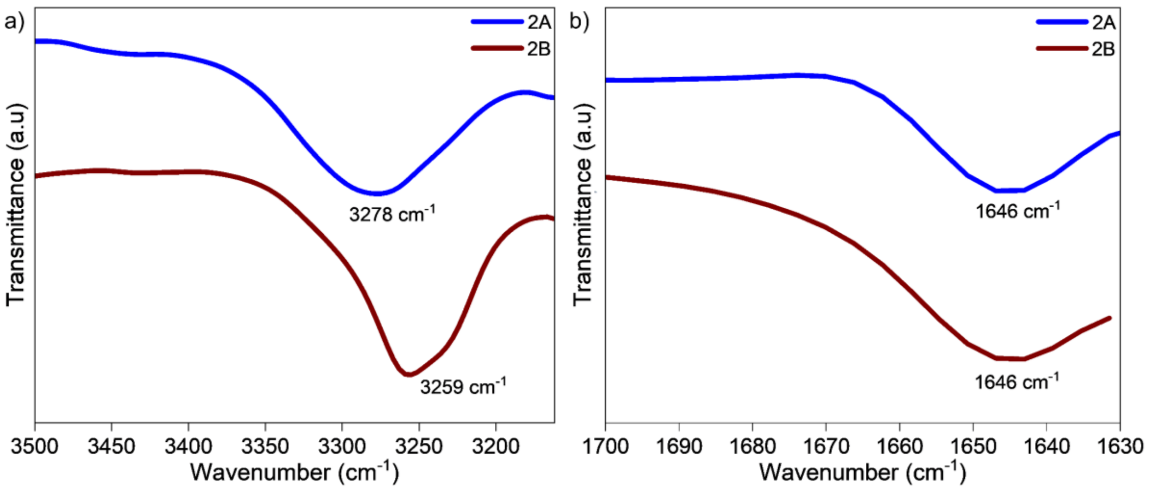

Figure S17: Partial FT-IR spectrum of aggregates **2A** and **2B** (MCH, *c* = 1 × 10^{-3} M) showing regions of a) N-H and b) carbonyl C=O stretching frequencies.

6. Morphological Studies

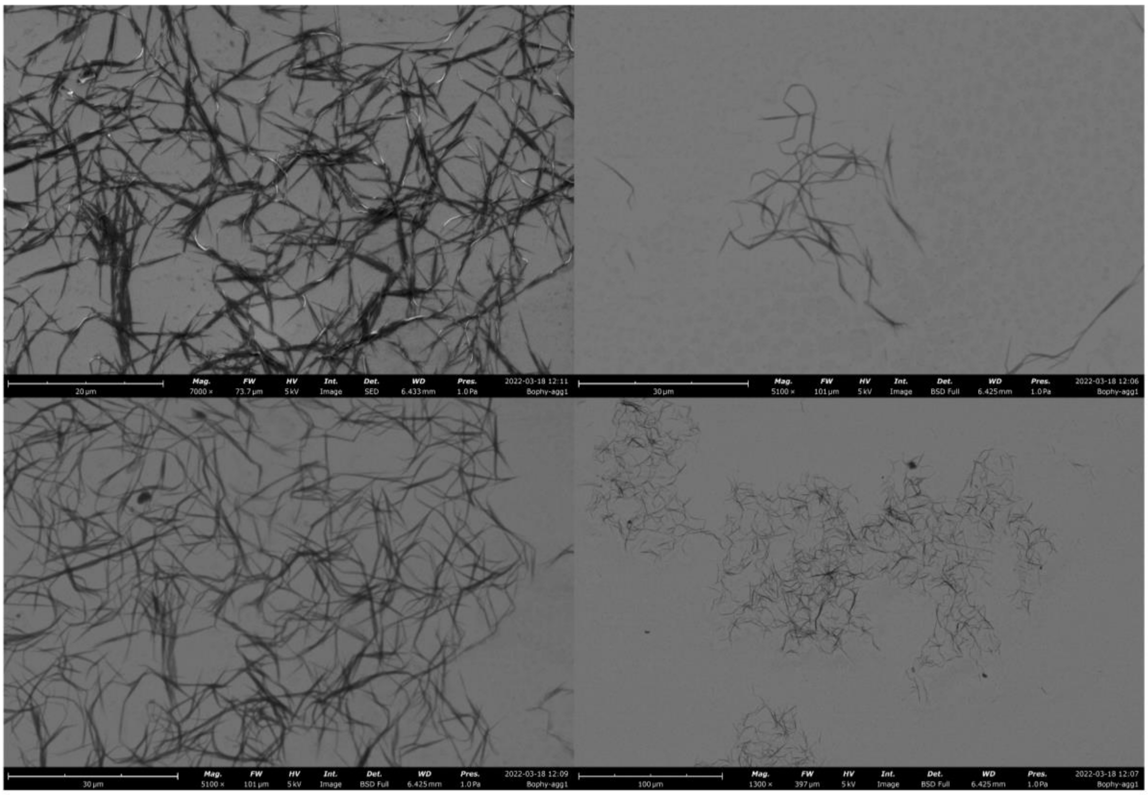

Figure S18: SEM images of aggregate **2A** prepared by drop-casting 10 µL of **2** (*c*= 10 µM in MCH) on a silicon wafer substrate. The images reveal elongated and fibre-like structures with moderate bundling.

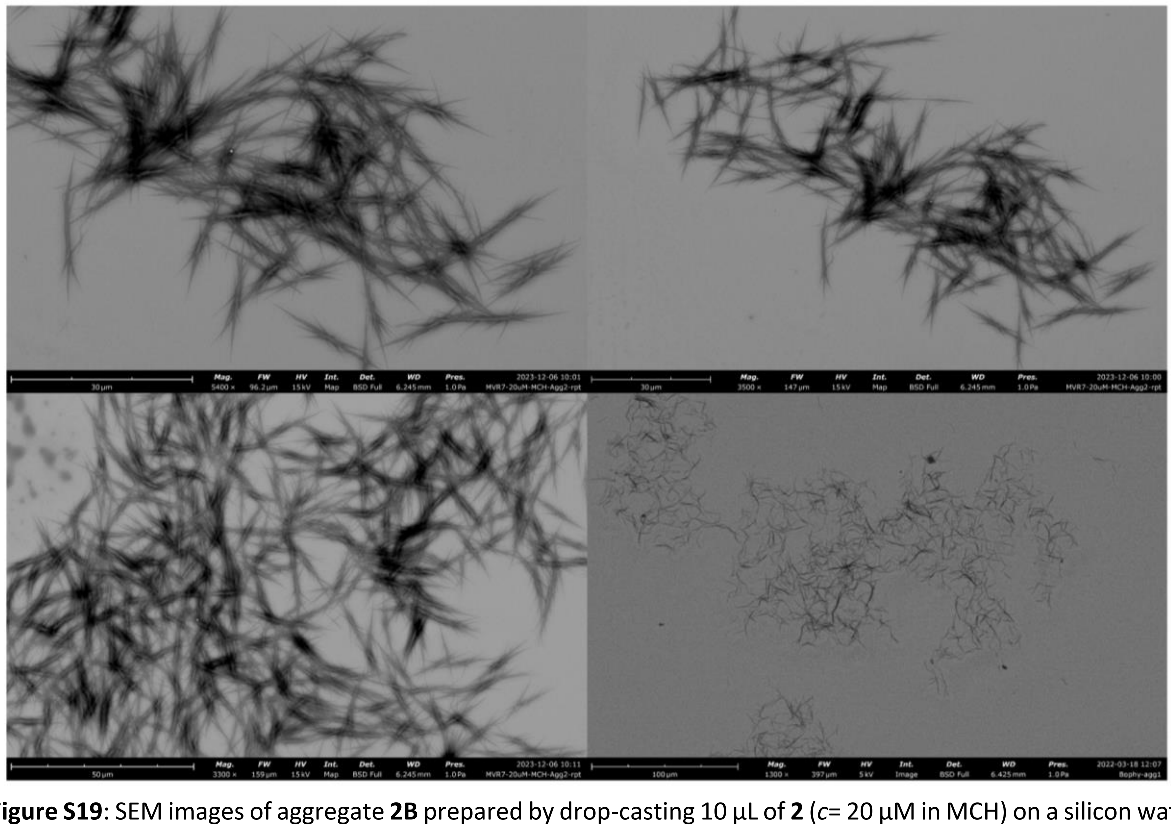

Figure S19: SEM images of aggregate **2B** prepared by drop-casting 10 µL of **2** (*c*= 20 µM in MCH) on a silicon wafer substrate. The images reveal elongated fibre-like structures with a more significant bundling than those of **2A**.

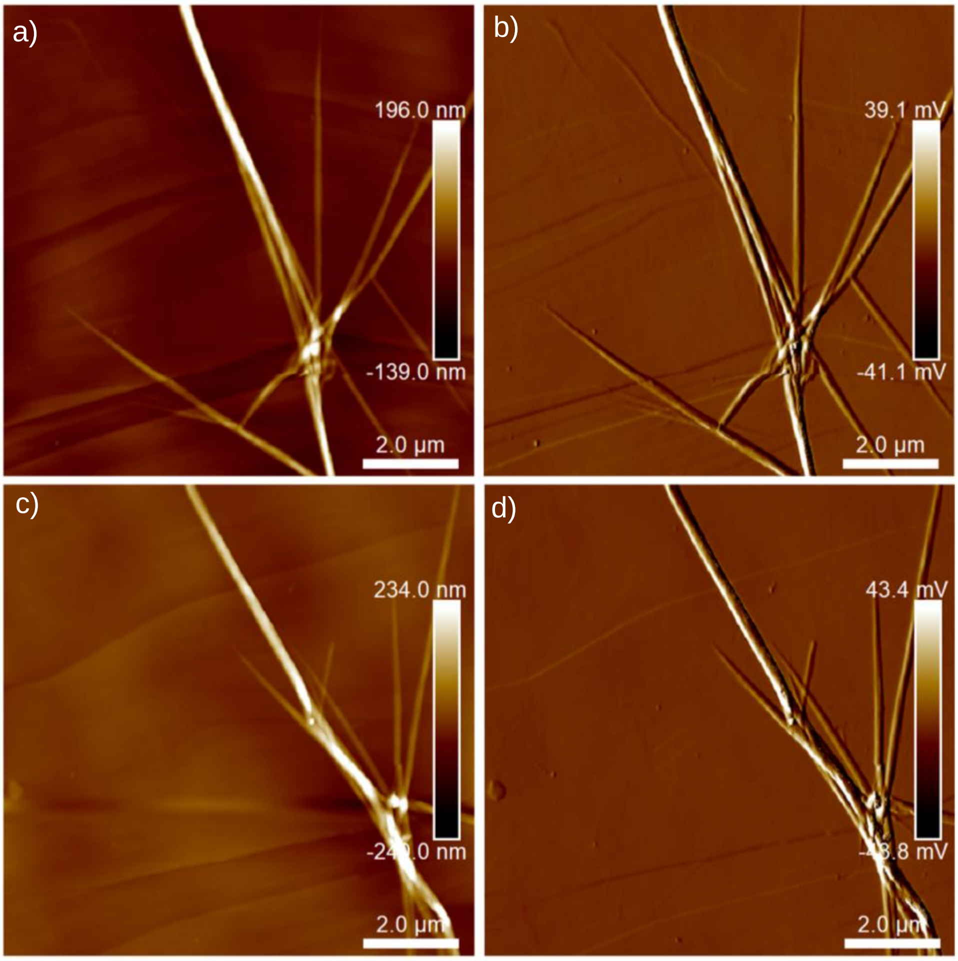

Figure S20: AFM height (a,c) and corresponding phase (b,d) images of aggregate **2A** prepared by cooling a 10 µM solution in MCH from 363 K to 298 K with a cooling rate of 1 K min^{-1} followed by drop-casting the sample on HOPG surface.

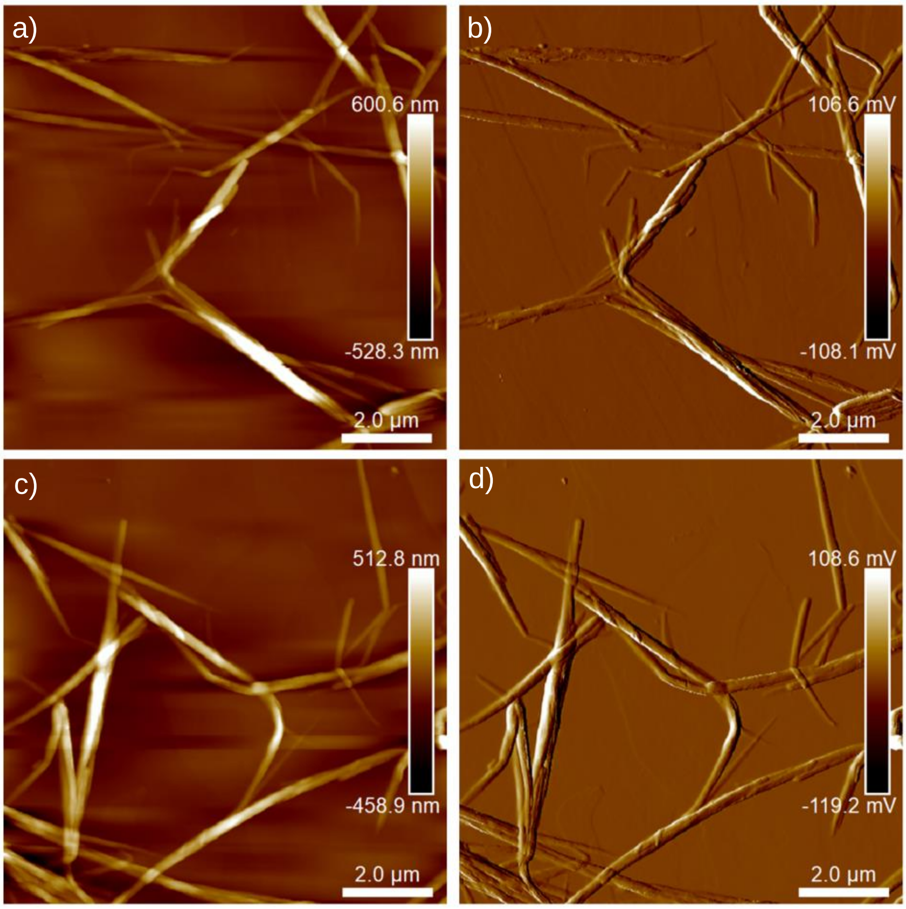

Figure S21: AFM height (a,c) and corresponding phase (b,d) images of aggregate **2B** prepared by ageing aggregate **2A** in MCH (c = 10 µM) for one day (24h) at room temperature (sonicating the sample for 5 seconds to break the larger precipitates) followed by drop-casting the sample on HOPG surface.

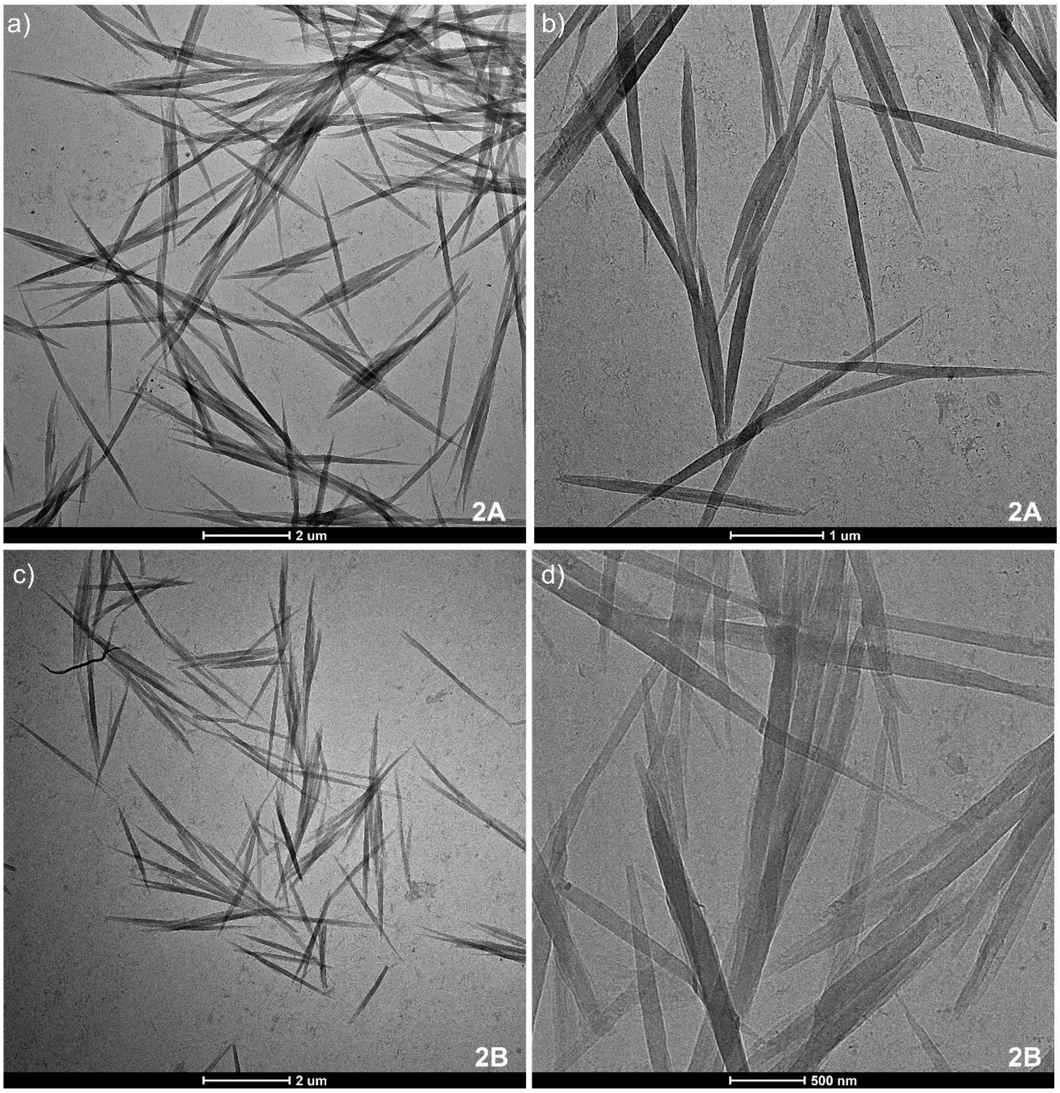

Figure S22: TEM images of **2B** (c,d) prepared by ageing aggregate **2A** (a,b) in MCH (c = 10 µM) for one day (24h) at room temperature (sonicating the sample for 5 seconds to break the larger precipitates) followed by drop-casting the sample on carbon coated mesh copper grid.

TEM measurements further confirm that the aggregates **2A** and **2B** also exhibit similar morphologies to those observed in AFM and SEM.

7. Theoretical Calculations

The DFT B3LYP/6-31g(d,p) basis set[7,8] was used to perform the geometry optimization of the different supramolecular species (monomer, dimers and trimers). To reduce the computational cost of theoretical calculations, the long alkoxy chains were replaced by methoxy groups. The corresponding absorption spectra for the monomer and trimers were calculated by using the rcam- B3LYP/6-31g(d,p) method. All computations were carried out using Gaussian-16 (G16RevC.01). [11] The time-dependent density functional theory (TD-DFT)[9] was selected for the geometry optimization (monomer, dimers and trimers), employing the CAM-B3LYP density functional[10] together with the 6-31G(d,p) basis set.[7, 8] The corresponding absorption spectra for the monomer and trimers were calculated by TD-DFT using the rcam-B3LYP/6-31g(d,p) method including 80 excitation energies. PyMOL was used as molecular visualization program.

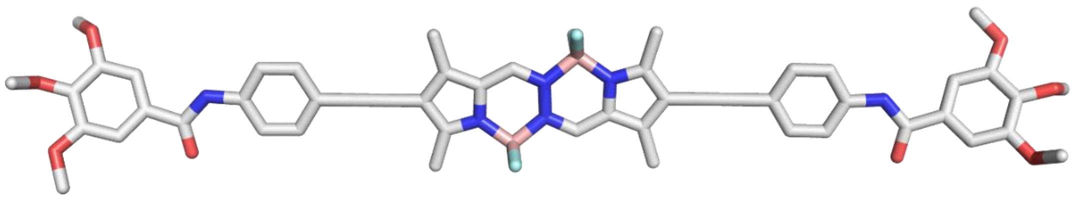

Figure S23: DFT B3LYP/6-31g(d,p) geometry-optimized monomer of molecule **2**.

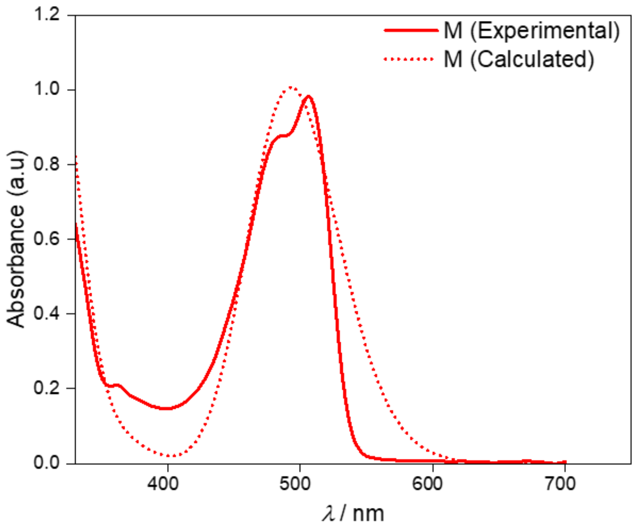

Figure S24: Calculated UV-Vis spectra for the (rcam- B3LYP/6-31g(d,p) method) monomer of molecule **2** and comparison with the experimental absorption spectrum in $CHCl_3$.

Side view

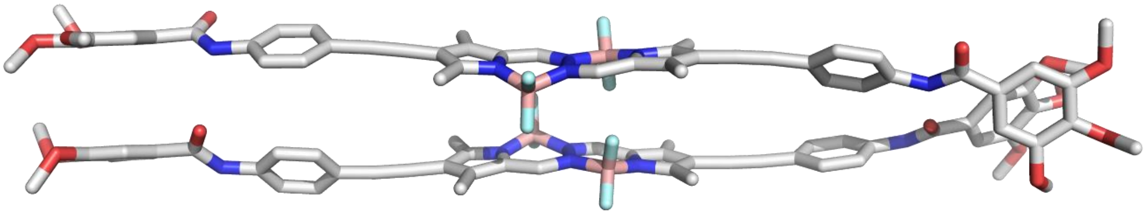

Top view

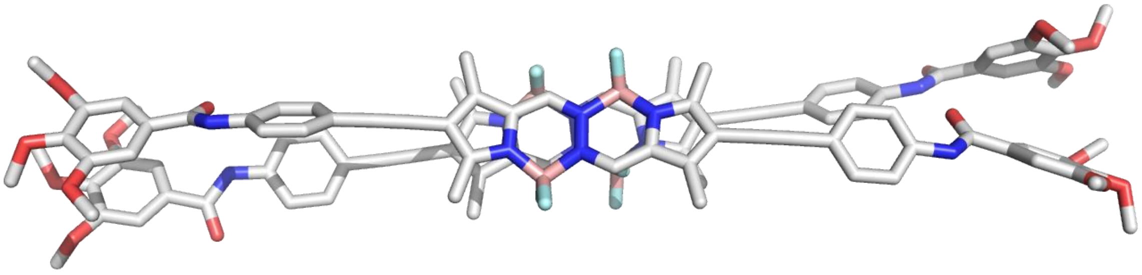

Figure S25: DFT B3LYP/6-31g(d,p) geometry-optimized dimer of aggregate **2A**.

Side view

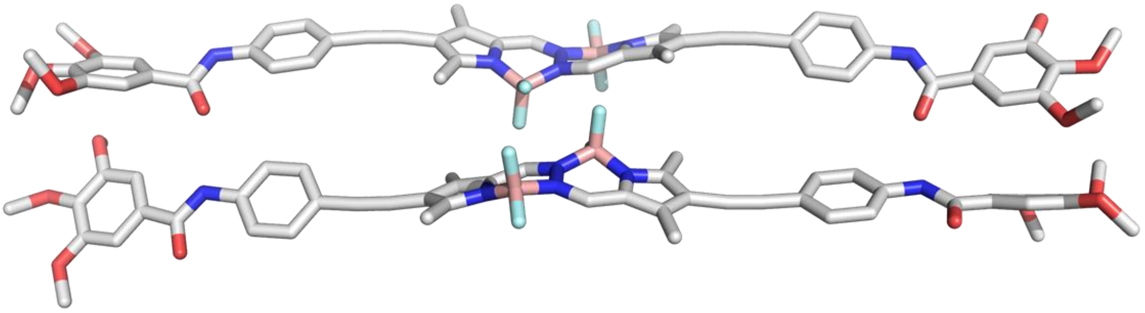

Top view

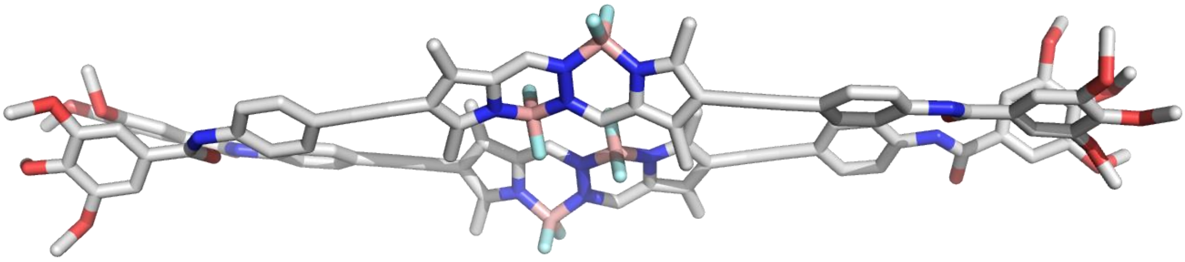

Figure S26: DFT B3LYP/6-31g(d,p) geometry-optimized dimer of aggregate **2B**.

Side view

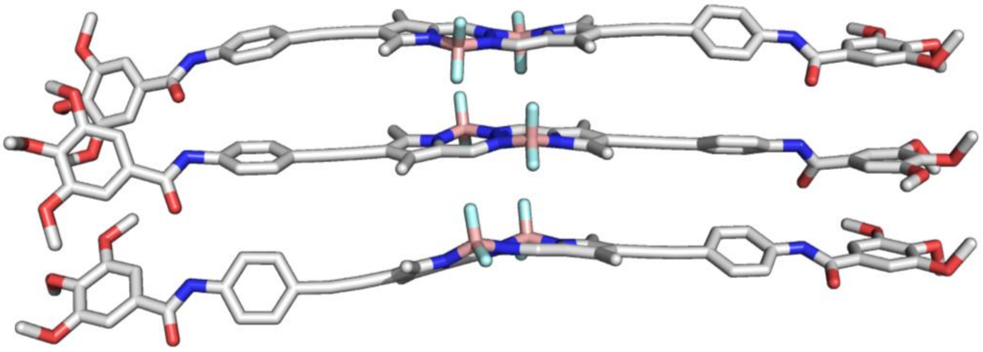

Top view

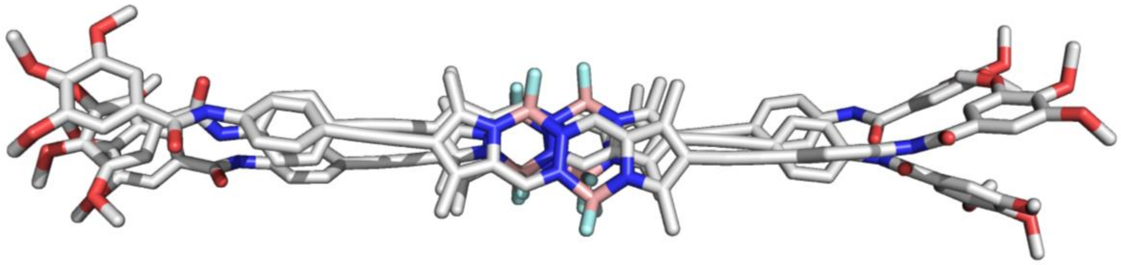

Figure S27: DFT B3LYP/6-31g(d,p) geometry-optimized trimer of aggregate **2A**.

Side view

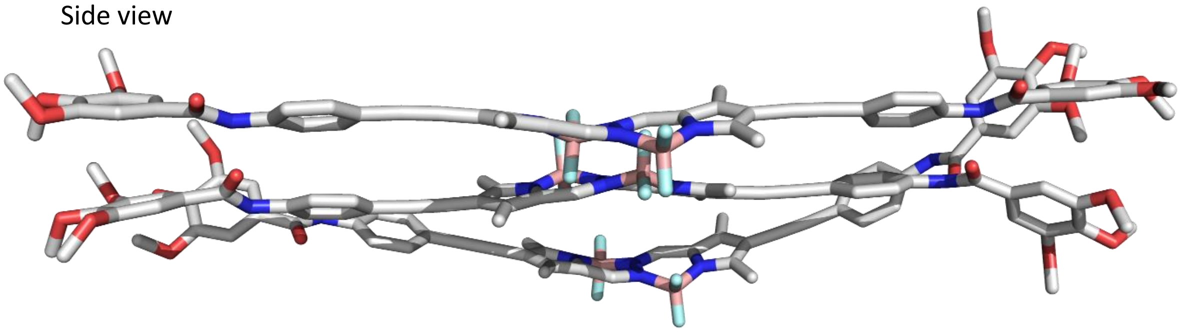

Top view

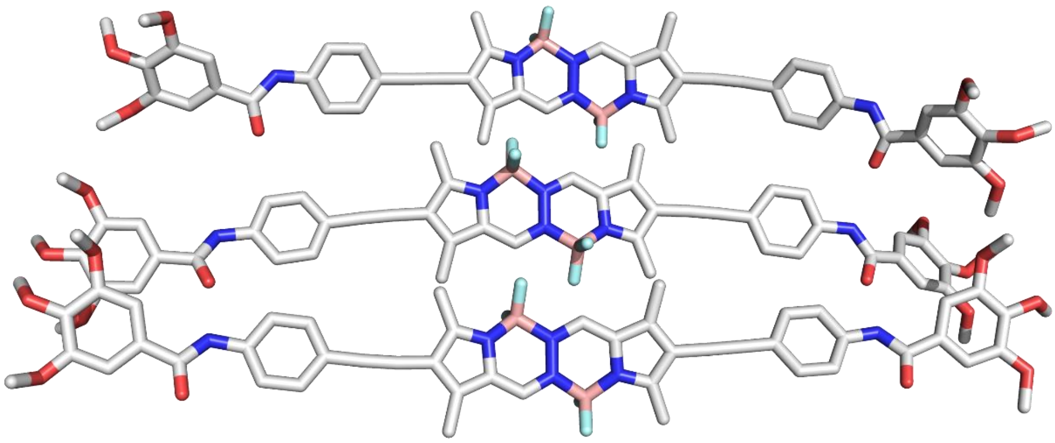

Figure S28: DFT B3LYP/6-31g(d,p) geometry-optimized trimer of aggregate **2B**.

Table S5: Different H-bond distances in aggregates **2A** and **2B**.

Aggregate	C-H···F-B distance (Å)	N-H···O=C distance (Å)
**2A**	2.468, 3.595	2.105, 2.263
**2B**	1.999, 1.967	1.992, 2.074

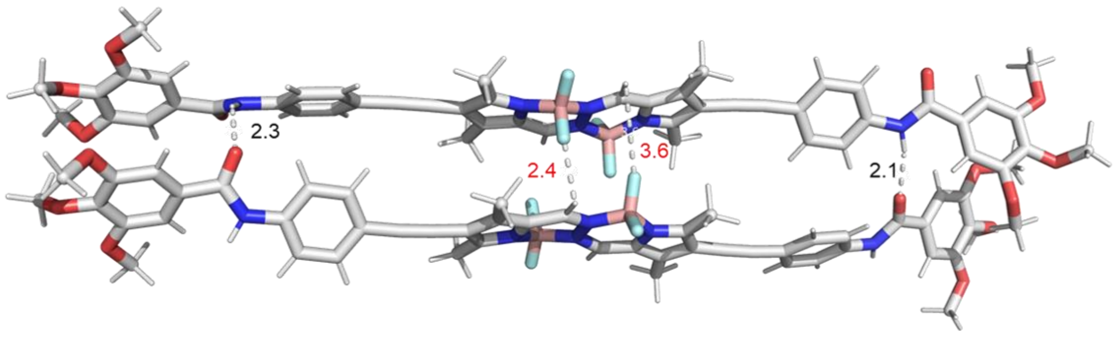

Figure S29: DFT B3LYP/6-31g(d,p) geometry-optimized dimer of aggregate **2A** along with C-H···F-B (marked with red numbers) and N-H···O=C (marked with black numbers) intermolecular H-bonding distances.

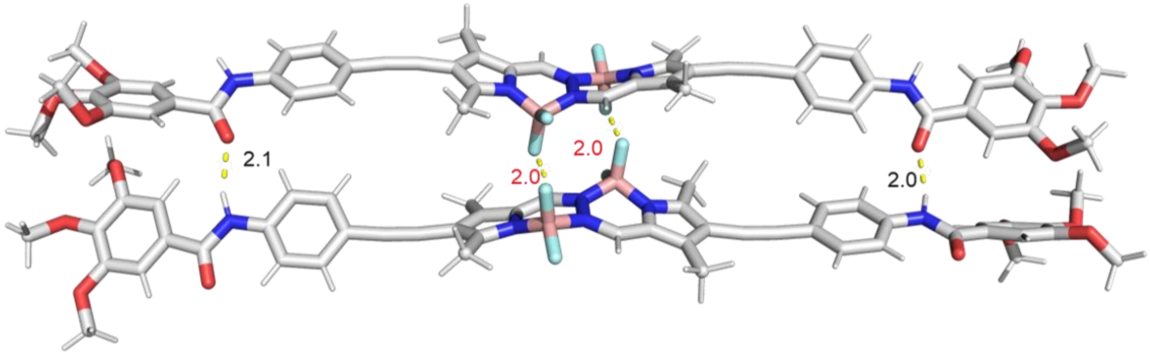

Figure S30: DFT B3LYP/6-31g(d,p) geometry-optimized trimer of aggregate **2B** along with C-H···F-B (marked with red numbers) and N-H---O=C (marked with black numbers) intermolecular H-bonding distances.

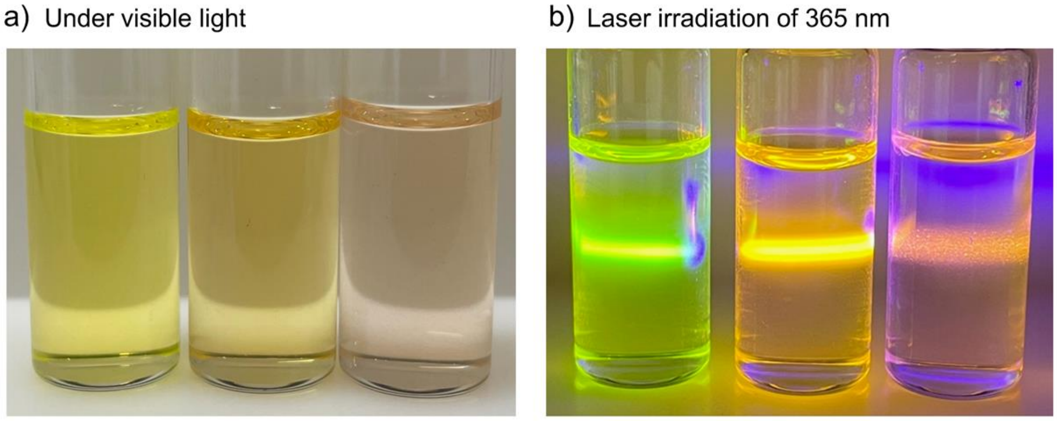

Figure S31: Photographs of monomer ($CHCl_3$), **2A** (MCH) and **2B** (MCH) under a) visible light and b) laser irradiation of 365 nm (c = 10 µM, 298 K). Monomer: left vial; **2A**: middle vial; **2B**: right vial.

The unusual emission features of the face-to-face stacked aggregate **2A** probably arise from the defects in the packing as also evident from the above-mentioned theoretical calculations.

REFERENCES

[1] Rödle, A.; Ritschel, B.; Mück-Lichtenfeld, C.; Stepanenko, V.; Fernández, G. *Chem.–Eur. J.*, **2016**, *22,* 15772–15777.

[2] Tamgho, I.-S.; Hasheminasab, A.; Engle, J.T.; Nemykin, V.N.; Ziegler, C.J. *J. Am. Chem. Soc*, **2014**, *136,* 5623–5626.

[3] Eikelder, H.M.M. ten; Markvoort, A.J.; Greef, T.F.A. de; Hilbers, P.A.J. *, J. Phys. Chem. B*, **2012**, *116,* 5291–5301.

[4] Markvoort, A.J.; Eikelder, H.M.M. ten; Hilbers, P.A.J.; Greef, T.F.A. de; Meijer, E.W. *Nat. Commun.*, **2011**, *2,* 509.

[5] Goldstein, R.F.; Stryer, L. *Biophys. J.*, **1986**, *50,* 583–599.

[6] Korevaar, P.A.; Schaefer, C.; Greef, T.F.A. de; Meijer, E.W. *J. Am. Chem. Soc*, **2012**, *134,* 13482–13491.

[7] Becke, A.D. *J. Chem. Phys*, **1993**, *98,* 5648–5652.

[8] Francl, M.M.; Pietro, W.J.; Hehre, W.J.; Binkley, J.S.; Gordon, M.S.; DeFrees, D.J.; Pople, J.A. *J. Chem. Phys*, **1982**, *77,* 3654–3665.

[9] Runge, E.; Gross, E.K.U. *Phys. Rev. Lett.*, **1984**, *52,* 997–1000.

[10] Yanai, T.; Tew, D.P.; Handy, N.C. *Chem. Phys. Lett.*, **2004**, *393,* 51–57.

[11] Gaussian 16, Revision B.01, Frisch, M.J., Trucks, G.W., Schlegel, H.B., Scuseria, G.E., Robb, M.A., Cheeseman, J.R.; Scalmani, G.; Barone, V.; Petersson, G.A.; Nakatsuji, H.; Li, X.; Caricato, M.; Marenich, A.V.; Bloino, J., Janesko, B.G., Gomperts, R., Mennucci, B., Hratchian, H.P., Ortiz, J.V., Izmaylov, A.F., Sonnenberg, J.L., Williams-Young, D., Ding, F., Lipparini, F., Egidi, F., Goings, J., Peng, B., Petrone, A., Henderson, T., Ranasinghe, D., Zakrzewski, V.G., Gao, J., Rega, N., Zheng, G., Liang, W., Hada, M., Ehara, M., Toyota, K., Fukuda, R., Hasegawa, J., Ishida, M., Nakajima, T., Honda, Y., Kitao, O., Nakai, H., Vreven, T., Throssell, K., Montgomery Jr., J.A., Peralta, J.E., Ogliaro, F., Bearpark, M.J., Heyd, J.J., Brothers, E.N., Kudin, K.N., Staroverov, V.N., Keith, T.A., Kobayashi, R., Normand, J., Raghavachari, K., Rendell, A.P., Burant, J.C., Iyengar, S.S., Tomasi, J., Cossi, M., Millam, J.M., Klene, M., Adamo, C., Cammi, R., Ochterski, J.W., Martin, R.L., Morokuma, K., Farkas, O., Foresman, J.B., Fox, D.J. Gaussian, Inc., Wallingford CT (2016) GaussView 5.0. Wallingford, E.U.A.
